# ISG15 Regulates Peritoneal Macrophages Functionality against Viral Infection

**DOI:** 10.1371/journal.ppat.1003632

**Published:** 2013-10-10

**Authors:** Emilio Yángüez, Alicia García-Culebras, Aldo Frau, Catalina Llompart, Klaus-Peter Knobeloch, Sylvia Gutierrez-Erlandsson, Adolfo García-Sastre, Mariano Esteban, Amelia Nieto, Susana Guerra

**Affiliations:** 1 Institute of Medical Virology, University of Zurich, Zurich, Switzerland; 2 Department of Preventive Medicine and Public Health, Universidad Autónoma, Madrid, Spain; 3 Department of Molecular and Cellular Biology, Centro Nacional de Biotecnología CSIC, Madrid, Spain; 4 Ciber de Enfermedades Respiratorias, Madrid, Spain; 5 Institute of Neuropathology, University Freiburg, Freiburg, Germany; 6 Department of Microbiology, Icahn School of Medicine at Mount Sinai, New York, New York, United States of America; 7 Department of Medicine, Division of Infectious Diseases, Icahn School of Medicine at Mount Sinai, New York, New York, United States of America; 8 Global Health and Emerging Pathogens Institute, Icahn School of Medicine at Mount Sinai, New York, New York, United States of America; University of Texas Southwestern Medical Center, United States of America

## Abstract

Upon viral infection, the production of type I interferon (IFN) and the subsequent upregulation of IFN stimulated genes (ISGs) generate an antiviral state with an important role in the activation of innate and adaptive host immune responses. The ubiquitin-like protein (UBL) ISG15 is a critical IFN-induced antiviral molecule that protects against several viral infections, but the mechanism by which ISG15 exerts its antiviral function is not completely understood. Here, we report that ISG15 plays an important role in the regulation of macrophage responses. ISG15−/− macrophages display reduced activation, phagocytic capacity and programmed cell death activation in response to vaccinia virus (VACV) infection. Moreover, peritoneal macrophages from mice lacking ISG15 are neither able to phagocyte infected cells nor to block viral infection in co-culture experiments with VACV-infected murine embryonic fibroblast (MEFs). This phenotype is independent of cytokine production and secretion, but clearly correlates with impaired activation of the protein kinase AKT in ISG15 knock-out (KO) macrophages. Altogether, these results indicate an essential role of ISG15 in the cellular immune antiviral response and point out that a better understanding of the antiviral responses triggered by ISG15 may lead to the development of therapies against important human pathogens.

## Author Summary

Modification of proteins by ubiquitin (UB) and ubiquitin-like proteins (UBLs) are key regulatory processes of the innate and adaptive immune response. Interferon (IFN) stimulated gene product 15 (ISG15) is an ubiquitin-like protein modifier, which is reversibly conjugated to different viral and cellular proteins mediating considerable antiviral responses. In turn, many viruses, including poxviruses, have evolved strategies to block the antiviral and inflammatory effects of the innate immune responses to keep cells alive until virus replication is completed. Here, we describe a novel function of ISG15 in the control of macrophages activation, phagocytosis and apoptosis in response to viral infection. These processes are essential for the self-defense mechanism to protect animals from infectious disease and could be crucial to understand the ISG15 antiviral activity described in animal models.

## Introduction

The host innate immune response represents a critical initial line of defense against invading pathogens, and the magnitude of this early response can influence the course of disease progression. One of the earliest host responses to viral infection is the production of type I interferon (IFN-α and-β) and the subsequent upregulation of IFN-stimulated genes (ISGs) [1], [2]. These ISGs generate an antiviral state and play an important role in determining the host innate and adaptive immune responses. One of the most highly induced genes in the IFN response is *ISG15*, which encodes a small UBL protein of 17 kDa that forms covalent conjugates with cellular proteins mediating considerable antiviral responses [3]. During viral infection in mice, ISG15 exists in three different forms: unconjugated within the cell, conjugated to target proteins and released into the serum [4]. When ISG15 is secreted, free ISG15 can function as a cytokine that modulates the immune response. For example, free ISG15 can activate natural killer (NK) and cytotoxic T-cells, stimulate IFN-γ production and induce dendritic cell maturation and neutrophil recruitment [5], [6]. In addition, antiviral activity associated with protein ISGylation has been described *in vitro* and/or *in vivo* for both DNA and RNA viruses, including influenza A and B viruses [7], Sindbis virus [7], [8], hepatitis B virus [9], herpes simplex type-1 virus [7], vaccinia virus [10], vesicular stomatitis virus [11], [12], lymphocytic choriomeningitis virus [13], respiratory syncytial virus [14], HIV-1 [15] and Ebola virus [16]. In contrast, free ISG15, but not ISGylation, promotes antiviral responses against Chikungunya virus infection [17].

Mice lacking UbE1L, the ISG15 E1 enzyme required for ISG15 conjugation, are more susceptible to influenza virus infection, indicating that ISGylation is essential for ISG15 antiviral activity [18]. ISG15 was shown to conjugate to over 160 host and viral proteins [19], especially those undergoing active translation [20]. Some of these host target proteins are downstream effectors of the interferon signaling, such as double-stranded RNA-activated protein kinase (PKR) [21], Myxovirus resistance protein A (MxA), and Retinoic acid inducible gene-I (RIG-I) [4], while others are involved in the regulation of type I IFN signaling, e.g. Janus kinase 1 (JAK1), extracellular signal-regulated kinase (ERK1), interferon regulatory transcription factor 3 (IRF3) and signal Transducers and Activator of transcription 1 (STAT1) [22]. The ISGylation of these cellular proteins may also contribute to the antiviral activity of ISG15. Moreover, it has been described that ISG15 expression blocks the virus-budding process by different mechanisms such the blockage of Endosomal Sorting Complexes Required for Transport (ESCRT) machinery for HIV [15], or in the case of Ebola and other enveloped viruses infections, inhibiting the Nedd4 E3 ubiquitin ligase [23]. Furthermore, several viral proteins have been shown to be conjugated to ISG15, such as the NS1 protein from influenza virus, NS5A from Hepatitis C virus [24] and gag from HIV virus [25]. It has been proposed that conjugation to viral proteins inhibits specific viral functions or virions assembly, causing a block in viral infection progression [26], [27]. Concerning VACV replication, previous publications from our group showed that ISG15 exerts antiviral activity against this virus, and that viral E3 protein can bind to ISG15, counteracting its activity. Thus, a VACV strain lacking E3 (VVΔE3L) was unable to replicate in ISG15+/+ cells, but was able to replicate in ISG15−/− deficient cells [10]. Moreover, infection of ISG15**−/−** mice with VVΔE3L resulted in significant disease and mortality, which was not observed in ISG15+/+ mice infected with this attenuated virus [10]. Besides the antiviral activity of ISG15, it has been recently described that a mutation in human ISG15 correlates with “Mendelian susceptibility to mycobacterial disease” (MSMD), a rare disorder that manifests in severe clinical symptoms following infection with weakly virulent mycobacterial strains and other intracellular pathogens [28].

Although the role of ISG15 as a defense molecule against the infection by several pathogens is accepted, the mechanisms by which its antiviral properties are exerted and the main cellular populations responsible for these activities are still weakly defined. In order to characterize whether the ISG15 antiviral activity observed in animal models is cell-type specific and to further characterize its implication in the immune response, we have now analyzed the impact of ISG15 deficiency on VACV replication using different primary cell lines (fibroblasts, dendritic cells and macrophages) derived from ISG15+/+ and ISG15**−/−** mice. Here, we have observed that macrophages are key effectors of ISG15-mediated antiviral responses during VACV infection and, very importantly, that one of the most essential functions of the macrophages, the phagocytosis, is dramatically diminished in the absence of ISG15. These results provide valuable information on the underlying mechanisms governing the suppression of viral infection by ISG15.

## Results

### ISG15−/− fibroblasts and macrophages respond differently to VACV infection

**Figure 1:**
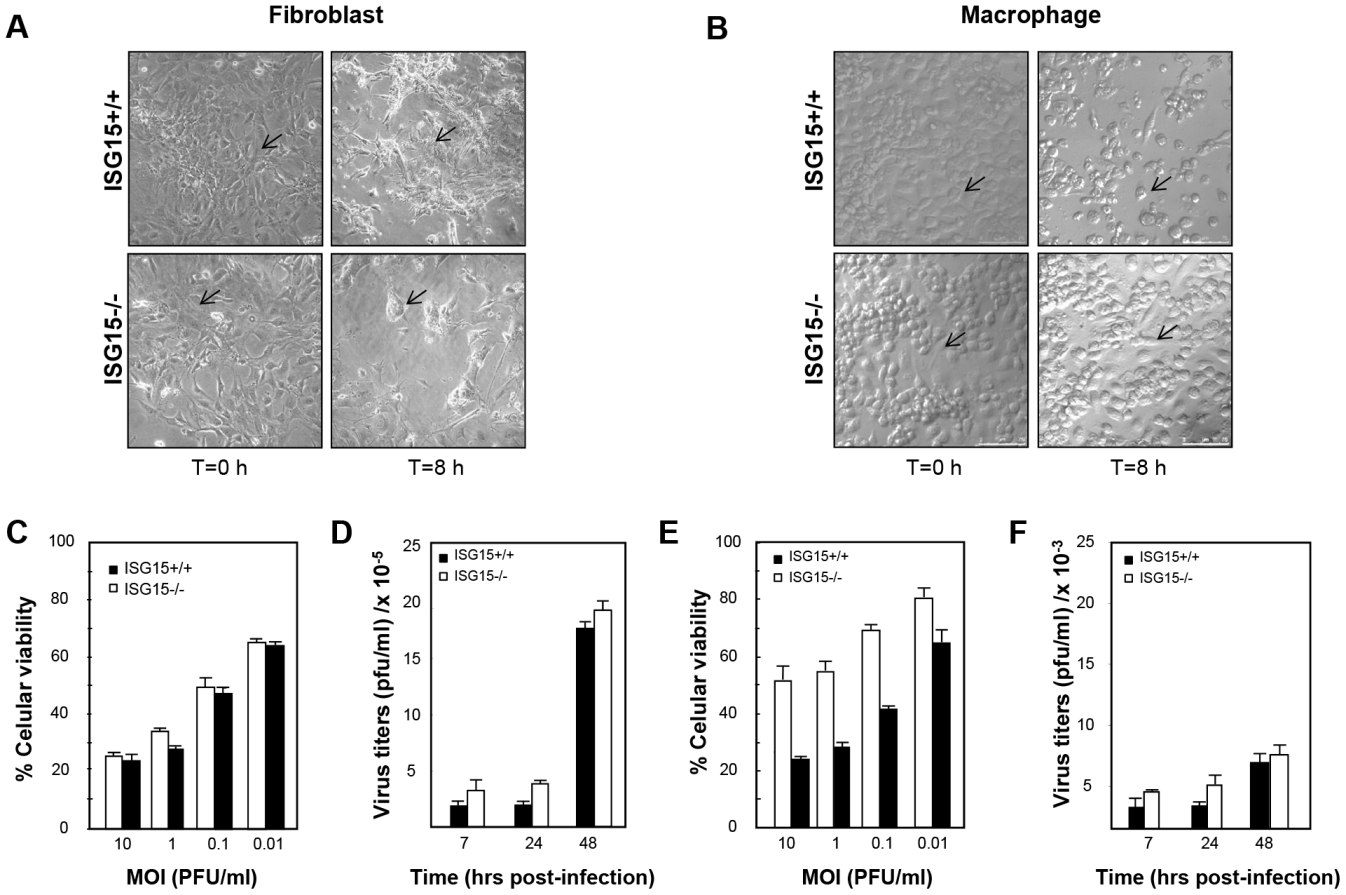
Effect of ISG15 on cytotoxicity and virus growth on MEFs or macrophages infected with VACV. **A–B.** ISG15+/+ or ISG15**−/−** MEFs (**A**) or peritoneal macrophages (**B**) were infected with VACV (WR strain, 5 PFU/cell) and CPE was visualized by phase-contrast microscopy at indicated times post-infection. A selected cell is indicated with an arrow. **C–E.** ISG15−/− or ISG15+/+ MEFs (**C**) or macrophages (**E**) were infected with VACV at the indicated MOI and, 24 hours post infection (hpi), cell viability was quantified as described in Materials and Methods. Results represent the mean ± the standard deviation of three independent experiments. P-values from a two-tailed t-test assuming non-equal variance were determined. In all the cases, P<0.05. **D–F.** One-step VACV growth on infected (0.1 PFU/cell) ISG15+/+ or ISG15−/− MEFs (**D**) or peritoneal macrophages (**F**). Cells were infected and, at the different times indicated, cells were harvested and virus yields were determined by plaque assay. Results represent the mean ± the standard deviation of three independent experiments. P values from a two-tailed t test assuming non-equal variance were determined. In all the cases, P<0.01.

**Figure 2:**
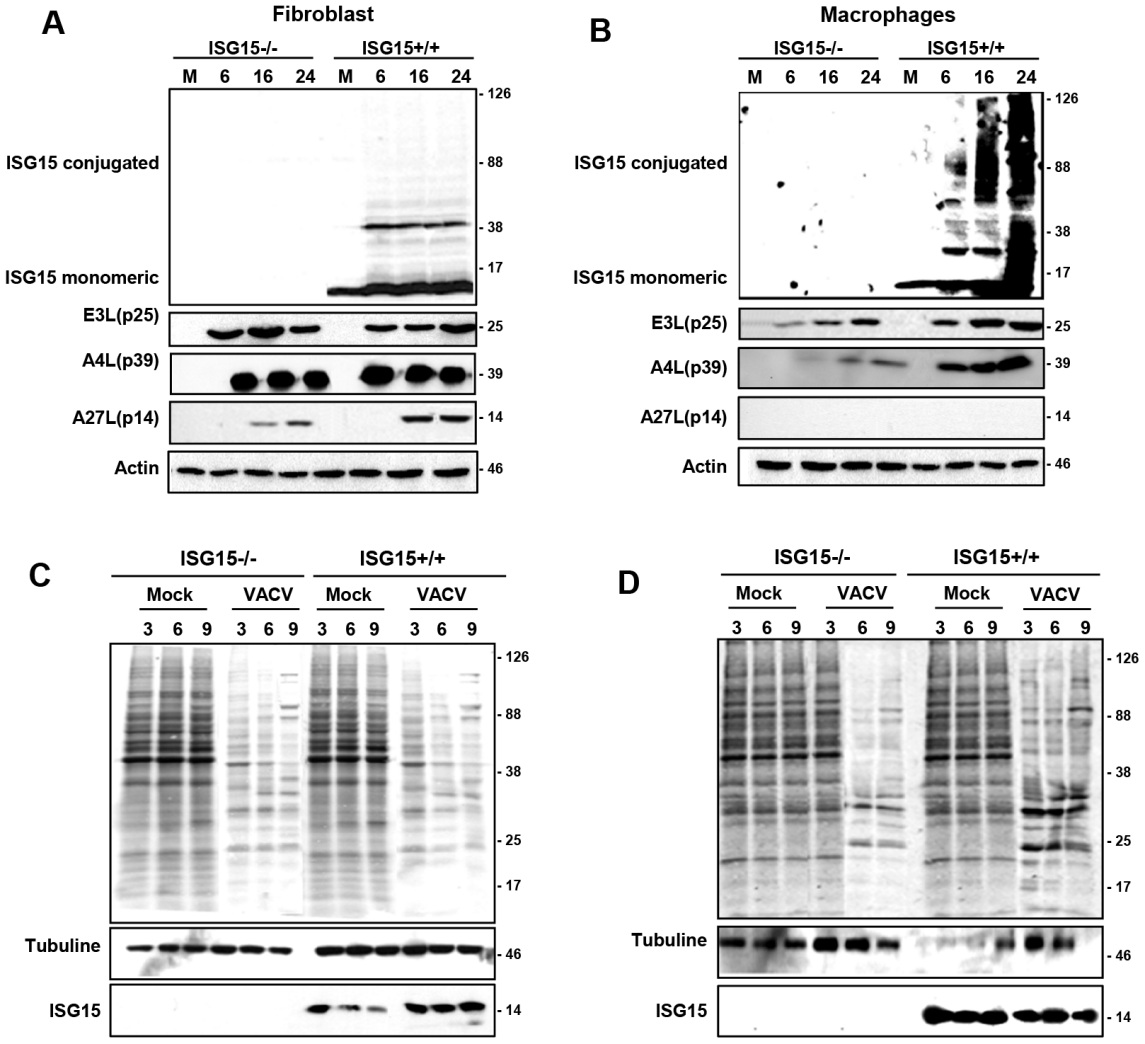
VACV protein synthesis on ISG15+/+ or ISG15−/− MEFs or macrophages. **A–B.** Viral protein expression during VACV infection of ISG15+/+ or ISG15−/− MEFs or macrophages. ISG15+/+ or ISG15−/− MEFs (**A**) or macrophages (**B**) were infected (10^6^ cells/time post-infection; 3 PFU/cell) with VACV (WR strain) and, at the indicated times post-infection, cell extracts were prepared and equal amounts of proteins were fractionated by SDS-PAGE, transferred to nitrocellulose, and detected with specific antibodies virus early (p25), intermediate (p39) and late proteins (p14). ISG15 expression levels were detected by Western blot using specific antibodies. Actin was measured as protein loading control. Protein standards are indicated. Uninfected cells (Mock) served as control. **C–D.** *De novo* protein synthesis in VACV-infected ISG15+/+ or ISG15−/− MEFs (**C**) or macrophages (**D**). ISG15+/+ or ISG15**−/−** cells were infected (10^6^ cells/time post-infection; 3 PFU/cell) with VACV (WR strain) and labeled with ^35^S-methionine (50 µCi/ml, 30 min) at the different times indicated. Cellular lysates were analyzed by 12% SDS-PAGE, transferred to nitrocellulose membranes and visualized by autoradiography. Protein standards are indicated. Uninfected cells (Mock) served as control. In the same membranes, the expression of ISG15 or tubuline (protein loading control) was detected by Western blot using specific antibodies.

In order to characterize if different ISG15 deficient cell types exhibit variable susceptibility to viral infection, we examined VACV replication in different primary cells, derived from both ISG15+/+ and ISG15**−/−** mice. As a first indicator, we analysed the cytopathic effect (CPE) induced in fibroblasts (MEFs) or peritoneal F4/F80 positives macrophages (Figure S1) after VACV infection (5 PFU/cell). CPE was estimated by cell rounding and alterations in cell morphology. While no differences in the VACV-induced CPE were observed between wild type and ISG15−/− MEFs (Fig. 1A), VACV-induced CPE was clearly detectable in ISG15+/+ macrophages (Fig. 1B and Movie S1), but not in ISG15−/− cells (Fig. 1B and Movie S2). In order to confirm these observations and to get a more quantitative result, we measured the cellular mortality produced by VACV infection in the different cell lines by a cellular viability assay. In agreement with the previous results, in the case of VACV-infected MEFs (Fig. 1C), no differences in the percentage of cellular viability at 24 hours post-infection were observed in any case and, as expected, percentage of cell death correlated with the multiplicity of infection used both in for ISG15+/+ and ISG15−/− cells. However, in the infected peritoneal macrophages (Fig. 1E), a higher percentage of ISG15−/− cells survived to the infection, at each multiplicity of infection (MOI) analyzed, in comparison to the results in ISG15+/+ macrophages. In order to evaluate if the differences in cell viability in response to VACV infection were due to variations in the viral production in the absence of ISG15, we quantified virus titers in both ISG15+/+ and ISG15**−/−** MEFs and peritoneal macrophages infected with VACV in the same conditions. Upon VACV-infection, no significant differences in viral titters were observed between ISG15+/+ and ISG15**−/−** MEFs. Furthermore, and as expected, the viral titers increased with time (Fig. 1D) and correlated with the observed increase in cellular mortality (Fig. 1C). In contrast, in macrophages from both ISG15**−/−** or ISG15+/+ mice, and according to previously published results [29], the infection with VACV was abortive and viral titers did not increase over time (Fig. 1F). In addition, viral protein synthesis in both VACV-infected ISG15+/+ and ISG15**−/−** MEFs and peritoneal macrophages was evaluated by Western blot using specific antibodies for early p25 (*E3L*), intermediate p39 (*A4*L), and late viral proteins p14 (*A27L*). Proteins encoded by *E3L* and *A4L* genes were efficiently detected in lysates of all the infected cells (Fig. 2A–B). However the relative levels were significantly lower in the ISG15−/− macrophages, suggesting that viral infection could be blocked in an early step in these cells. Regarding the late proteins encoded by *A27L* genes, they were detected only in lysates from infected MEFs (Fig. 2A) and not in infected macrophages (Fig. 2B), confirming that VACV infection is abortive in macrophages. To further characterize these results, the kinetics of viral protein synthesis and the viral induced shut-off, as indicators of the infection progression, were evaluated by metabolic labeling at 3, 6 and 9 h after VACV infection in the different cell lines. In ISG15+/+ and ISG15**−/−** MEFs, the viral protein synthesis pattern presented similar kinetics, strongly suggesting that ISG15 does not significantly alter VACV replication in this cell type (Fig. 2C). However, in macrophages, kinetics of viral gene expression was strongly affected by the lack of ISG15. Whereas in ISG15+/+ macrophages, the synthesis of viral proteins was clearly detectable at 3 hpi, a clear delay in the course of infection and viral protein synthesis was observed in ISG15**−/−** macrophages. To further analyse the putative cell-specific role of ISG15 on viral replication, we carried out similar experiments in ISG15+/+ and ISG15**−/−** bone marrow**-**derived dendritic cells (BMDC) (data not shown). As observed in MEFs, similar levels of viral protein were expressed in ISG15+/+ and ISG15**−/−** BMDC. The above stated results could be explained by differences in ISG15 expression levels among MEFs and macrophages in response to viral infection. To evaluate this hypothesis, we analyzed the ISG15 expression levels (both non-conjugated and conjugated to its cellular protein target), which was clearly higher in VACV-infected macrophages than in the infected-MEFs (Fig. 2A–B). In summary, VAVC infection appears to be similar in ISG15+/+ and ISG15−/− MEFs, but appears to be reduced in ISG15−/− macrophages, which correlates with lower cell virus-induced death. Although these data appear counterintuitive, as ISG15 would have been expected to inhibit viral infection, and not to promote viral infection, these results suggest that ISG15 expression might be crucial for macrophage activation in response to viral infection and allow us to speculate that ISG15 could modify host factors involved in viral cycle modulation in macrophages.

### ISG15 controls apoptosis after VACV infection in peritoneal macrophages

**Figure 3:**
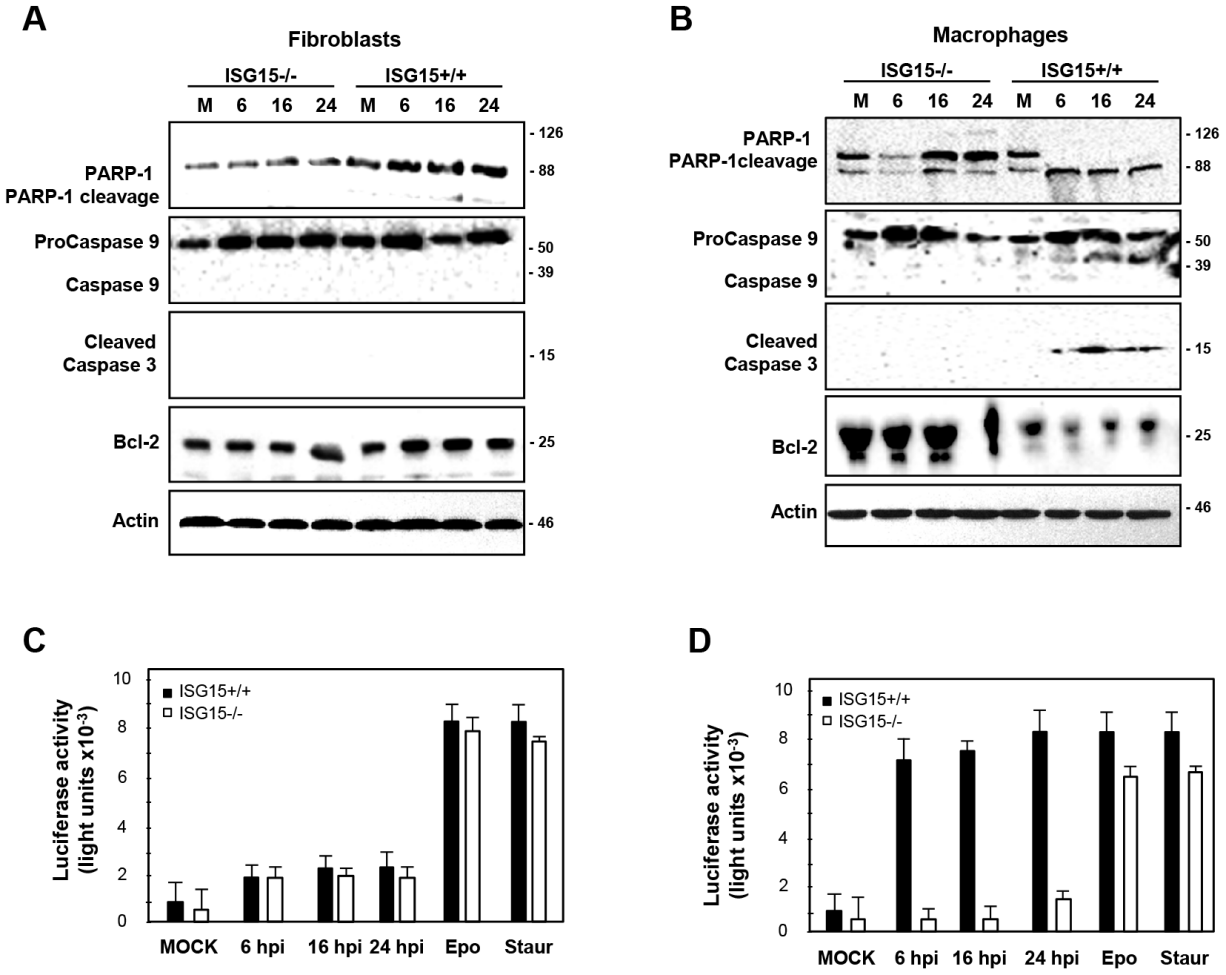
ISG15 controls apoptosis in macrophages after VACV infections. **A–B.** Time course of apoptosis markers during VACV infection in ISG15+/+ or ISG15**−/−** MEFs (**A**) or peritoneal macrophages (**B**). ISG15+/+ or ISG15−/− cells were mock-infected (M) or infected with VACV (WR strain, 10 PFU/cell). At the indicated times post-infection, cells were harvested and total proteins were separated by SDS-PAGE, transferred to nitrocellulose and immunoblotted with anti-PARP-1, anti-caspase-9, anti-activated-capsase-3 and anti bcl-2 specific antibodies. Actin was measured as protein loading control. Protein standards are indicated. **C–D.** Apoptosis activation in ISG15+/+ or ISG15−/− MEFs (**C**) or peritoneal macrophages (**D**). Apoptosis was measured using the Caspase-Glo 3/7 assay kit at 24 hpi Results represent the mean ± the standard deviation of three independent experiments. P-values from a two-tailed t test assuming non-equal variance were determined. In all the cases, P<0.05.

The above exposed results indicated that the infection of VACV is modulated in macrophages by an uncharacterized ISG15 dependent mechanism. Moreover, clear differences in viral-induced cell death were observed in these cells in the absence of ISG15, indicating that ISG15 could regulate macrophages programmed cell death in response to viral infection. To analyze this hypothesis, we first evaluated cell death by apoptosis in response to VACV infection, by the cleavage of the poly (ADP-ribose) polymerase-1 (PARP-1) using an AB that recognizes both full-length and cleaved forms of PARP-1 [30]. In infected MEFs, derived either from ISG15+/+ or from ISG15−/− mice, despite cell death (Fig. 1A), no evident signs of apoptosis were observed (Fig. 3A). However, an 89 kDa PARP-1 cleavage product, indicative of activation of the apoptotic cascade, was clearly observed at 6 hpi in ISG15+/+ macrophages. Remarkably, this fragment was only weakly detectable in the ISG15−/− infected macrophages (Fig. 3B), suggesting that activation of apoptosis was impaired in infected macrophages in in the absence of ISG15. To further confirm these observations, additional apoptosis activator markers were evaluated in ISG15+/+ and ISG15**−/−** MEFs or peritoneal macrophages infected with VACV. A clear activation of caspase 3 and 9 was only observed in ISG15+/+ infected macrophages, which correlated with lower levels of the anti-apoptotic factor B-cell lymphoma 2 (Bcl-2). In order to get a more quantitative and physiological indicator of the apoptosis activation in these cells, the Caspase-Glo 3/7 assay kit was used following manufacturer's instructions. As shown in Fig. 3C, VACV-induced apoptosis was almost undetected in ISG15+/+ and ISG15**−/−** MEFs, further validating the observations described above. However, when cells were treated with the apoptosis activators epopside and staurosporine, apoptosis in ISG15+/+ and ISG15**−/−** treated**-**MEFs reached similar levels, confirming that ISG15 does not play any role in the activation of the apoptosis cascade in MEFs. In contrast, VACV-induced apoptosis activation was only detected in ISG15+/+ cells but not in macrophages lacking ISG15. In order to exclude that ISG15 generally regulates macrophage apoptosis rather than controlling apoptosis induction upon viral infection, we again used the general apoptosis activators epopside and staurosporine. A slight diminution of the apoptosis induced by these compounds was detected in the absence of ISG15, indicating that, although it could be implicated in general apoptosis, ISG15 mainly regulates the apoptosis in response to viral infection (Fig. 3D). Although these data might be explained by a higher level of viral infection and viral protein synthesis in ISG15+/+ macrophages, resulting in enhanced apoptosis as compared to ISG15−/− macrophages, these results also suggest that in macrophages but not in MEFs ISG15 is involved in the specific activation of programmed cell death upon VACV infection.

### ISG15 regulates viral infection in macrophages

**Figure 4:**
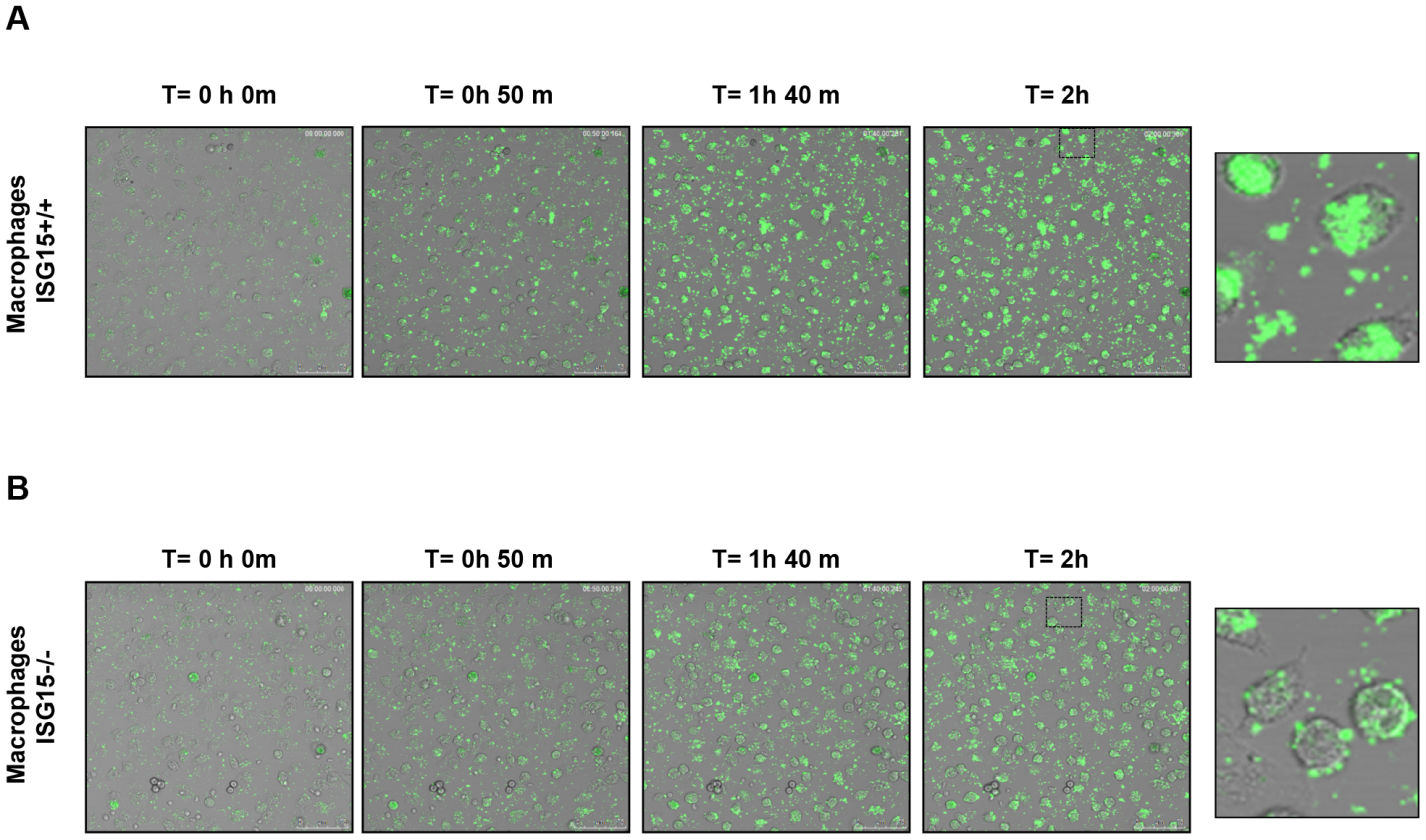
ISG15 control the VACV entry. Peritoneal macrophages isolated from ISG15+/+ or ISG15**−/−** mice were seeded in 8-well tissue culture plates and treated with type I interferon (IFN) (100units/ml) for 16 hours. After that, the cells infected with VACV-YFP (60 PFU/cell). Infected-cells were visualized with the time by fluorescent and phase contrast microscopy. Representative fields are shown at a magnification of 40× (left panels).

**Figure 5:**
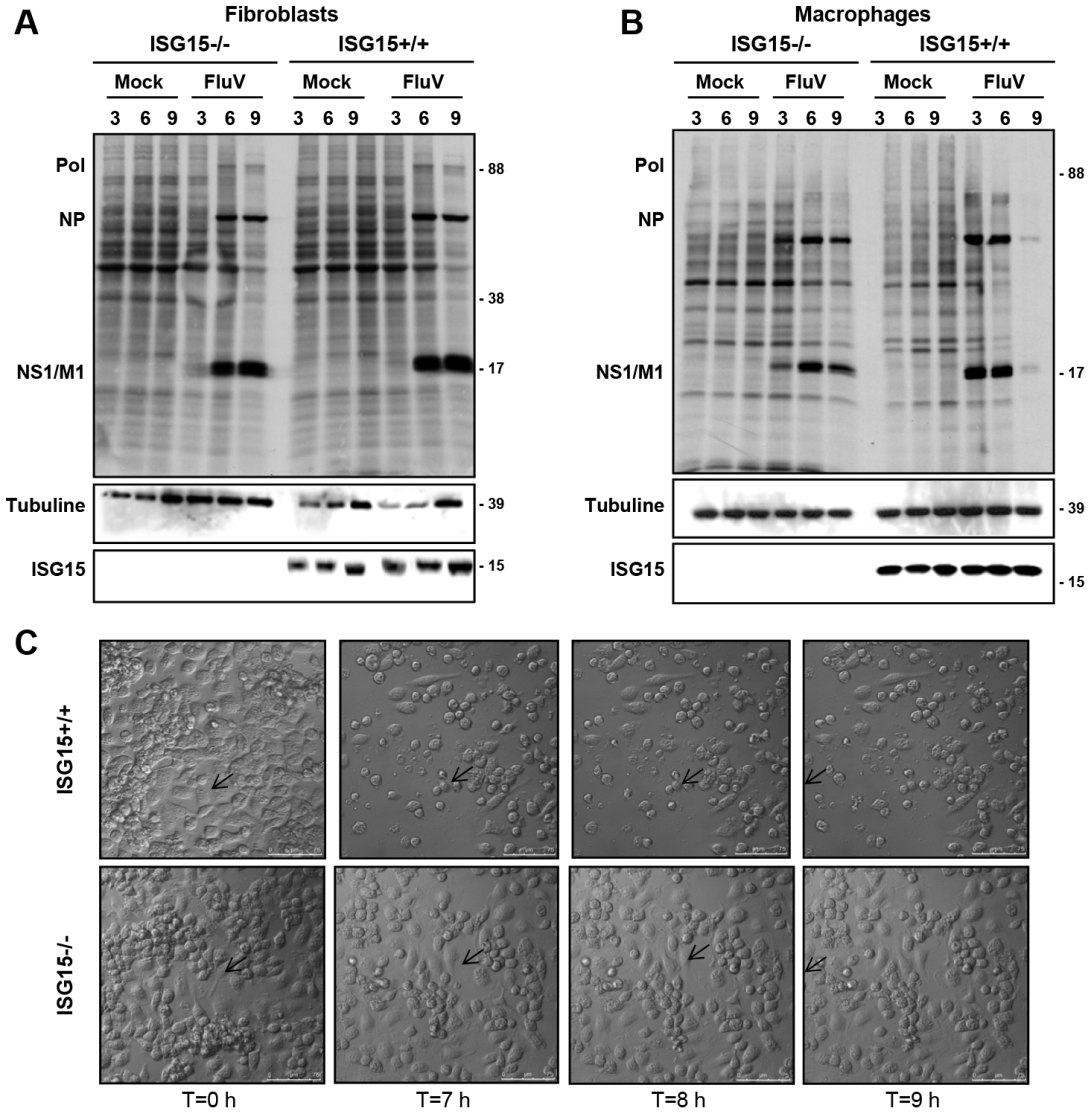
Effect of ISG15 on cytotoxicity and infection progression in ISG15+/+ or ISG15−/− MEFs or macrophages infected with influenza virus (FluV). **A–B.** *De novo* protein synthesis in FluV- infected ISG15+/+ or ISG15−/− MEFs (**A**) or peritoneal macrophages (**B**). ISG15+/+or ISG15**−/−** cells were infected (10^6^ cells/time post-infection; 3 PFU/cell) with FluV (A/*WSN*/1933 strain) and labeled with ^35^S-methionine (50 µCi/ml, 30 min) at the times indicated. Cellular lysates were analyzed by 12% SDS-PAGE, transferred to nitrocellulose membranes and visualized by autoradiography. Protein standards are indicated. Uninfected cells (Mock) served as control. In the same membranes, the expression of ISG15 or tubuline (protein loading control) was detected by Western blot using specific antibodies. **C.** ISG15+/+ or ISG15**−/−** peritoneal macrophages were infected with FluV (A/*WSN*/1933 strain, 5 PFU/cell) and CPE was visualized by phase-contrast microscopy at indicated times post-infection. A selected cell is indicated with an arrow.

The data presented above suggest that the virus infection itself could be the signal that triggers apoptosis in macrophages, as the VACV cycle appears to be prematurely inhibited in the absence of ISG15 in these cells. Given that ISG15−/− infected macrophages showed an increase in cellular survival, accompanied by a reduction in viral protein levels and a delayed kinetic of viral protein synthesis, we decided to analyze whether VACV entry was impaired in ISG15−/− macrophages. For that, we used a recombinant virus in which the viral structural protein A3L was labeled with the yellow fluorescent protein (YFP), allowing the visualization of the viral particles by time-lapse microscopy. When macrophages were infected with the VACV-YFP, we observed a delay in the entry of the virions inside the cell in the absence of ISG15 (Fig. 4). While in wild type (WT) macrophages the fluorescent signal increased with time inside the cell (Fig. 4A), in ISG15−/− macrophages, the signal was localized mainly outside the cell even after 2 hours of adsorption (Fig. 4B). These results indicate that, in the absence of ISG15, infection was blocked at an early step, which might also be the reason for the delay in the kinetics observed in the viral protein radiolabeling experiment (Fig. 2D). These results suggest that, in the absence of ISG15, the virus entry is impaired and made us to consider whether this mechanism was exclusive for VACV or if it also observed upon infection with other viruses. Therefore, we performed similar experiments using FluV, a completely different virus with different cellular receptor. In a first approach, we infected ISG15+/+ and ISG15**−/−** MEFs or peritoneal macrophages with FluV (5 PFU/cell) and, as above described for VACV, evaluated *de novo* viral protein synthesis using ^35^S-Met at different times post-infection. As observed for VACV infection, there are no variations in viral protein synthesis in MEFs in the absence of ISG15 (Fig. 5A), but a clear delay in the overall viral protein synthesis is observed in the infected ISG15**−/−** macrophages (Fig. 5B, compare 3 hpi in ISG15−/− and ISG15 +/+ macrophages). Although no viral production was observed in both ISG15+/+ and ISG15**−/−** macrophages (Figure S2), when we visualized the effect of FluV infection by phase**-**contrast microscopy, a clear CPE was evident in the ISG15+/+ macrophages with the course of infection, whereas the ISG15−/− macrophages remained unaltered with no obvious signs of viral infection-induced cell death (Fig. 5C). Since the block in infection and the differences in viral induced CPE were observed with two very distinct viruses, we next investigated whether other cellular entry processes, such as phagocytosis, might be regulated by ISG15 in macrophages.

### ISG15 regulates macrophage phagocytosis

**Figure 6:**
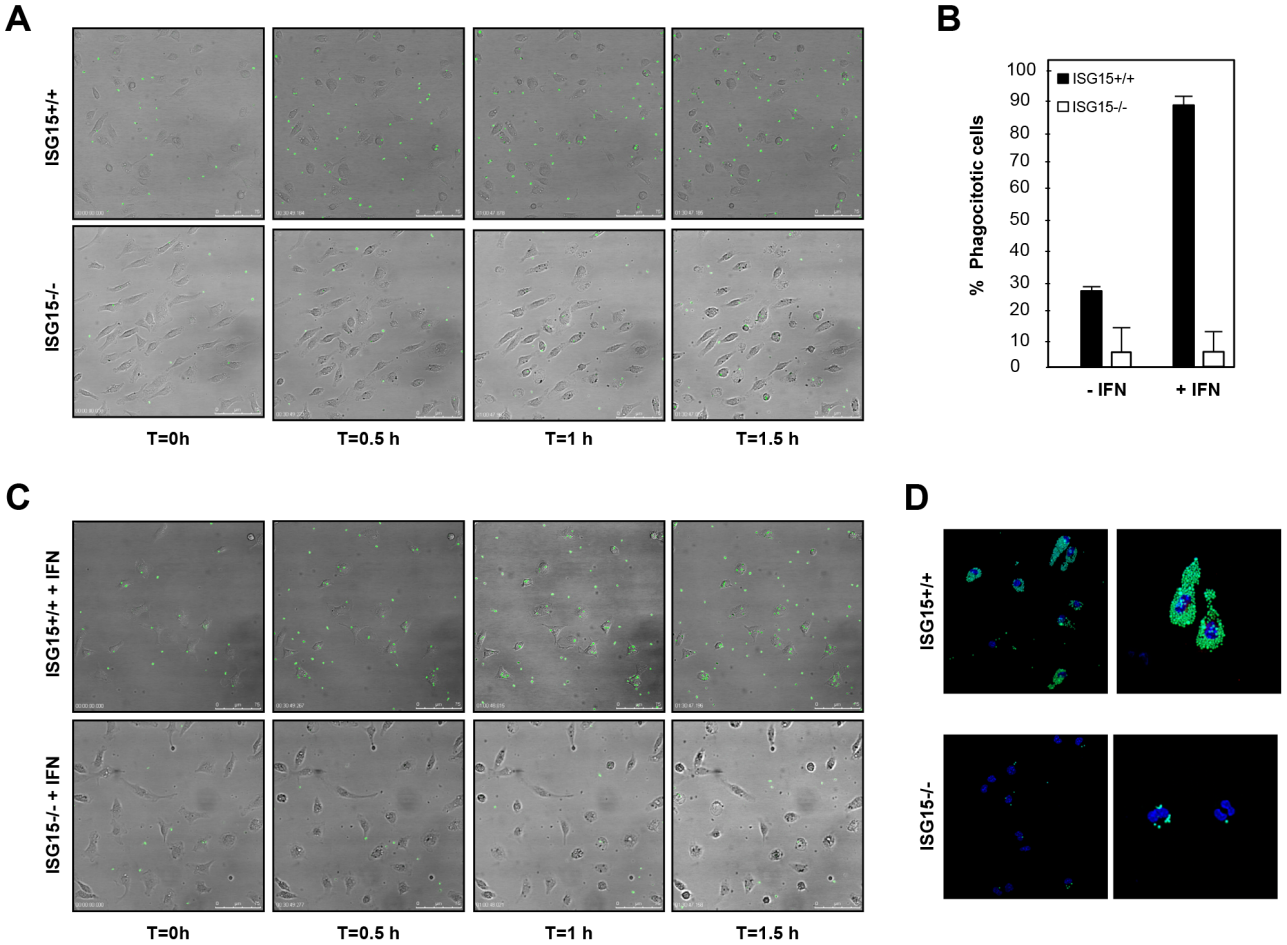
Phagocytosis of GFP-latex beads by ISG15 +/+ or ISG15 −/− macrophages. **A–C.** Peritoneal macrophages isolated for ISG15+/+ or ISG15**−/−** mice were seeded in 8-well tissue culture plates and treated with type I IFN (100units/ml) for 16 hours (**A**) or not (**C**). After that, the cells were incubated with 1**-**µm-diameter latex beads conjugated to GFP in a ratio of 10 latex beads per cell. Phagocytized beads and cells were visualized with the time by fluorescent and phase contrast microscopy. **B.** Quantification of phagocytic cells in type I IFN alpha treated or not treated macrophages. Cells were quantified by IF in three independent experiments. Black bars represent ISG15+/+ macrophages, white bars represents ISG15**−/−** macrophages. Results represent the mean ± the standard deviation of three independent experiments. P values from a two-tailed t-test assuming non-equal variance were determined. In all the cases, P<0.05. **D.** Phagocytosis of GFP-latex beads by ISG15+/+ and ISG15**−/−** macrophages detected by immunofluorescence. Peritoneal macrophages were cultured on coverslips and incubated for 1 h with GFP-latex beads (20 beads per cell) and then washed three times with phosphate-buffered saline (PBS) and incubated with Dulbecco's medium (DMEM) medium for an additional hour. Cells were fixed with 4% PFA and processed for immunofluorescence analysis. Phagocytized beads and cells were visualized by confocal fluorescent microscopy. Representative fields are shown at a magnification of 40× (left panels) and 100× (right panels).

A major function of macrophages is the phagocytosis of pathogens, antigens, and infected or apoptotic cells, which is critical for innate as well as for adaptive immunity. Taking into account that in ISG15−/− macrophages the early events in the infection cycle of two completely different viruses, VACV or FLuV, were aborted, and that this was at the level of virus endocytosis, at least for VACV, we considered that other cellular entry processes inherent to macrophage function, such as phagocytosis, could be controlled by ISG15. Latex beads are a common model substrate in biochemical studies of macrophage phagosome composition and maturation [31]. To determine whether ISG15 is critical for macrophage phagocytosis capacity, we analyzed the intake of GFP labelled latex beads in ISG15+/+ and ISG15**−/−** macrophages by confocal and time-lapse microscopy, as described in Material and Methods. To potentially enhance the impact of ISG15, which is an IFN induced protein, macrophages were treated with type I IFN alpha (100 units/ml for 16 hours) or left untreated [32]. The time-lapse microscopy images showed a marked decrease of latex beads phagocytosis in ISG15**−/−** macrophages when compared to the ISG15+/+ cells. This difference was even more evident upon IFN treatment, further implicating a role of ISG15 in this process (Fig. 6A–C and Movie S3-S4-S5-S6). Quantification of these results revealed that, after IFN incubation, the phagocytic capacity of ISG15+/+ cells was about 100 times higher than that observed in ISG15**−/−** cells (Figs. 6B). Moreover, and pointing out the biological relevance of ISG15 in phagocytosis, IFN treatment increased the latex bead uptake in ISG15+/+ macrophages but not in ISG15**−/−** cells (Fig. 6B). Representative confocal immunofluorescence images of macrophages with internalized beads are shown in Fig. 6D. Furthermore, to check if the treatment with IFN also enhance the entry of VACV in macrophages, we monitored by immunofluorescence the amount of virus inside the cell after 2 hpi in permeabilized ISG15+/+ and ISG15−/− cells treated or not with IFN. A clear increase of viral entry was observed after IFN treatment in ISG15+/+ infected macrophages (Figure S3). All together, these results strongly suggest that ISG15 has an important role in the phagocytic activity of macrophages that is in correspondence with an increase of viral entry, and that IFN enhances both processes through the induction of ISG15.

### ISG15 plays a critical role in AKT induced phagocytosis

**Figure 7:**
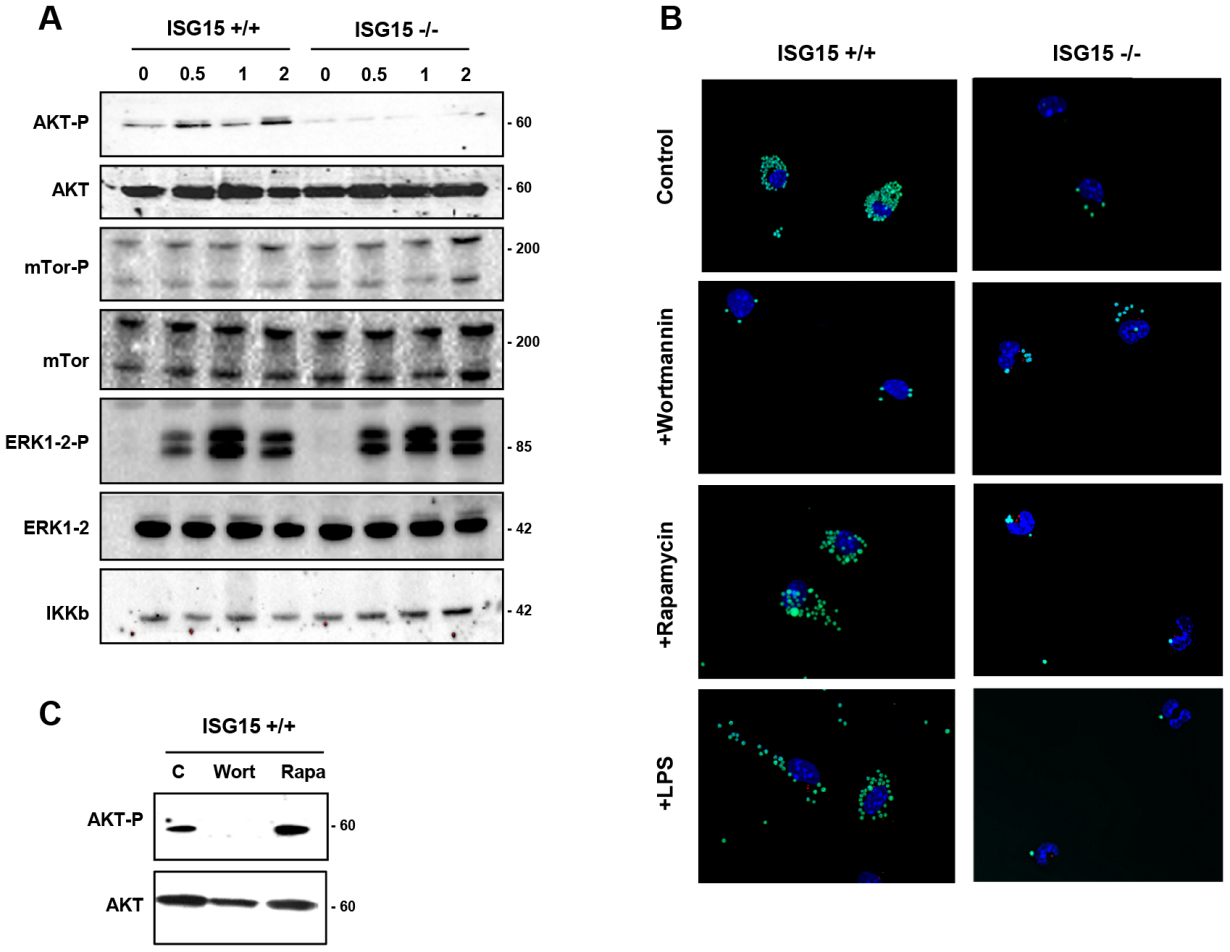
AKT phosphorylation levels are altered in ISG15+/+ or ISG15−/− macrophages after VACV infection. **A.** ISG15+/+ or ISG15**−/−** macrophages were infected with VACV (WR strain, 60 PFU/cel) and, at the indicated times, cells were harvested and total proteins were separated by SDS-PAGE, transferred to nitrocellulose and immunoblotted with anti-phosphoS473**-**AKT. The expression of total AKT was evaluated using a specific antibody (AB). **B.** Phagocytosis of GFP-latex beads by macrophages treated with wortmannin, rapamycin or LPS by confocal microscopy. Peritoneal macrophages isolated from ISG15+/+ or ISG15−/− mice were seeded in 8-well tissue culture plates and treated with type I IFN (100 units/ml) for 16 hours. After that, cells were untreated or treated with wortmannin (100 nM) or rapamycin (100 nM) and, 1 hour after the treatment, the cells were incubated with 1**-**µm-diameter latex beads conjugated to GFP in a ratio of 20 latex beads per cell. Phagocytized beads and cells were visualized with the time by confocal fluorescent microscopy. **C.** AKT phosphorylation levels after the treatment with PI3K pathway inhibitors. ISG15+/+ macrophages were untreated or treated with wortmannin (100 nM) or rapamicyn (100 nM) and, 1 hour after the treatment, cells were incubated with latex bead (10 bead per cell) for 2 hours. Cells were consecutively harvested and total proteins were separated by SDS-PAGE, transferred to nitrocellulose and immunoblotted with anti-phosphoSer473**-**AKT. The expression of AKT was evaluated using a specific antibody.

**Figure 8:**
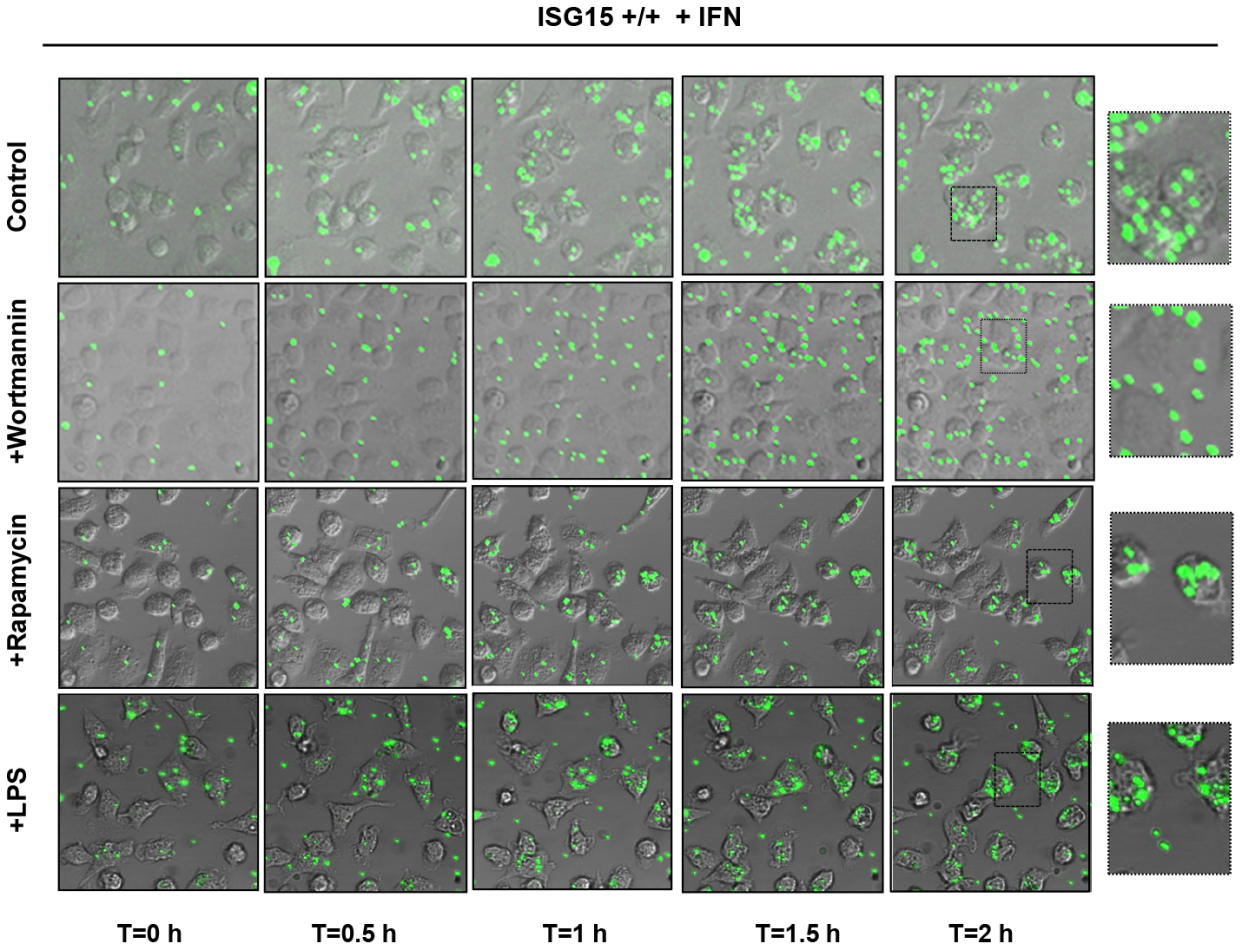
Wortmannin treatment inhibits phagocytosis of GFP-latex beads by ISG15+/+ macrophages. Peritoneal macrophages isolated from ISG15+/+ mice were seeded in 8-well tissue culture plates and treated with type I IFN (100 units/ml) for 16 hours. After that, were untreated or treated with wortmannin (100 nM) or rapamycin (100 nM) for 1 hour and subsequently incubated with 1**-**µm-diameter latex beads conjugated to GFP in a ratio of 20 latex beads per cell. Phagocytized beads and cells were visualized with the time by fluorescent and phase contrast microscopy. Representative fields are shown at a magnification of 40× (left panels) and 100× (right panels).

The activation of Phosphoinositide 3-kinase/(PI3K)- A protein-serine/threonine kinase (AKT) signaling has previously been shown to be required for macrophage phagocytosis [31], [33]. Therefore, we examined whether the activation of this signaling cascade could be affected in the absence of ISG15. The phosphorylation of AKT, mammalian Target of Rapamycin (m-Tor) and ERK1-2 in ISG15+/+ and ISG15−/− macrophages in response to VACV infection was monitored by Western blot. ISG15+/+ macrophages have some basal levels of AKT phosphorylation under the used conditions, which were increased at early times upon exposure to VACV. By contrast, basal levels of P-AKT were clearly reduced in ISG15**−/−** macrophages, when compared to ISG15+/+ cells, and these did not increased upon exposure to VACV (Fig. 7A). In contrast, phosphorylation levels of m-Tor or ERK1-2 were similar in both types of cell populations. Similar results were obtained when macrophages were incubated with latex beads (data not shown), indicating that the underlying ISG15-dependent mechanism of phagocytosis/endocytosis activation by both particles and viruses could be similar and related to AKT. To analyze whether the reduction in AKT phosphorylation observed in ISG15**−/−** macrophages is involved in the phagocytosis blockage, the phagocytosis capacity of WT macrophages was analyzed by confocal immunofluorescence using different inhibitors of the PI3K pathway. Treatment of IFN-exposed ISG15+/+ macrophages with wortmannin, an inhibitor of AKT phosphorylation (Fig. 7C), considerably reduced their phagocytic capacity (Fig. 7B and Movie S8) in comparison to untreated cells (Fig. 7B and Movie S7). In contrast, the mTOR inhibitor rapamycin, which preserves AKT phosphorylation (Fig. 7C), had no effect on the phagocytosis activity. We also checked if treatment with lipopolysaccharide (LPS), a well characterized macrophage activator, affected phagocytosis in both ISG15−/− and ISG15+/+ macrophages (without previous interferon treatment). The WT macrophages activated with LPS showed similar phagocytosis levels as controls (Fig. 7B and Movie S10). Strikingly, in ISG15−/− macrophages, no activation of phagocytosis was observed after LPS treatment (Fig. 7B). In addition to the confocal analyses, time-lapse microscopy was performed and representative images of the different treatments are shown in Figure 8. Only wortmanin treatment clearly reduced phagocytosis capacity of ISG15+/+ macrophages, as indicated by the high number of non-internalized and surface-bound beads in these cells. Collectively, these results indicate that ISG15 plays a critical role in AKT-phosphorylation, and that this pathway is essential to ensure proper phagocytosis in macrophages. These findings describe a novel function of ISG15 promoting macrophage phagocytosis.

### ISG15 promotes the antiviral activity of macrophages and the phagocytosis of VACV-infected cells

**Figure 9:**
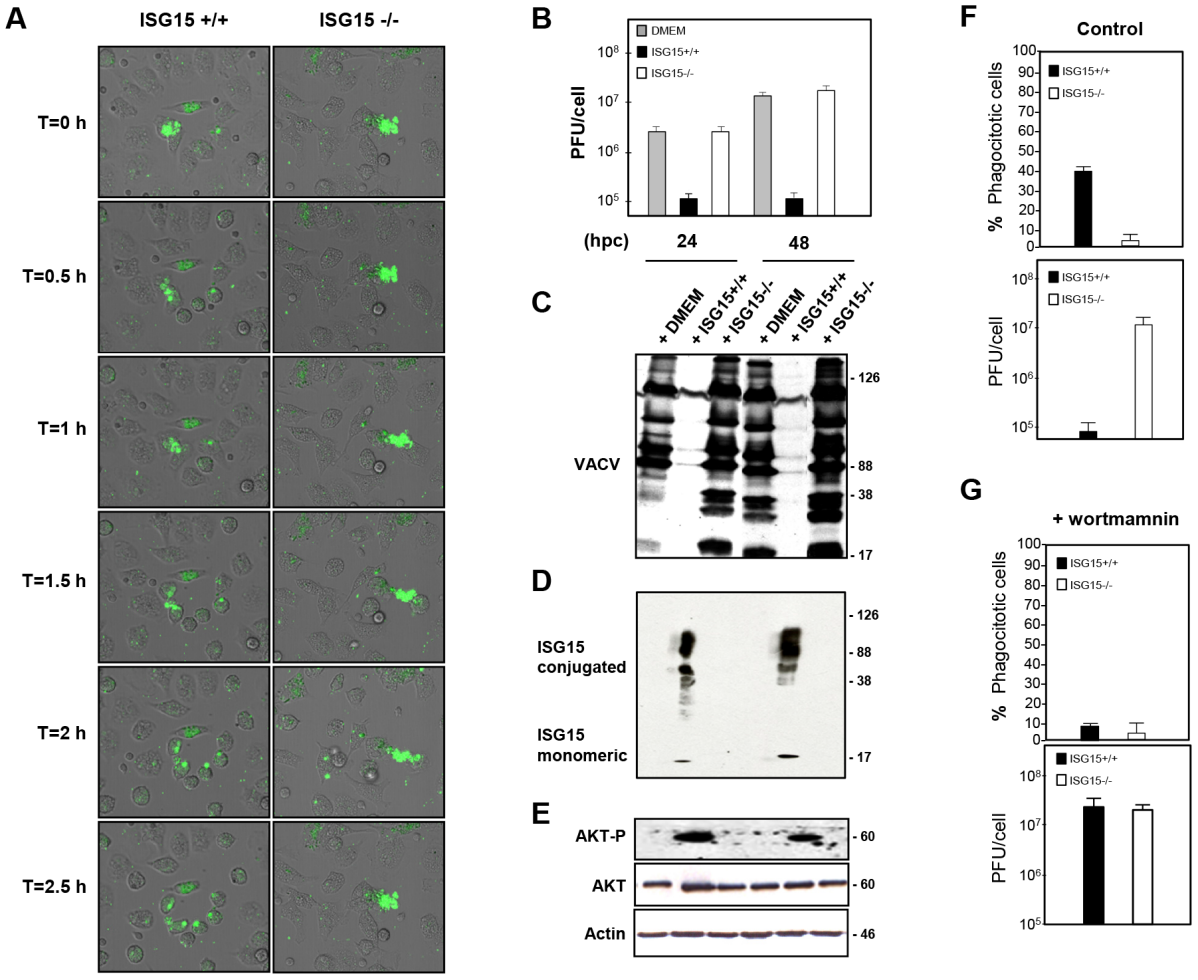
ISG15 regulates the phagocytosis and the clearance of VACV-infected MEFS regulating AKT phosphorylation. **A.** ISG15−/− MEFS were infected with VACV-YFP at 1 PFU/cell for 8 hours, and added to the ISG15+/+ or ISG15−/− macrophages culture with a ratio of one MEF cells to four macrophages. The mixed culture was monitored for 2,5 hours at 37°C by time-lapse microscopy and representative images were illustrated. **B.** Inhibition of VACV growth in the presence of ISG15+/+ macrophages. (A) ISG15−/− MEFS were infected with VACV-YFP at 1 PFU/cell for 8 hours and further cultured with DMEM (as negative control) or macrophages (ISG15+/+ or ISG15**−/−**) with a ratio of 1∶2 using 10^5^ macrophages. Infected cells were harvested at different times postinfection and virus yields were determined by plaque assay. Results represent the mean ± the standard deviation of three independent experiments. P values from a two-tailed t test assuming non-equal variance were determined. In all the cases, P<0.05. **C-D-E.** Viral protein expression (**C**) ISG15 (**D**) and AKT phosphorylation levels (**E**) were measured in the above described co-cultures. Total proteins from cells described above were separated by SDS-PAGE, transferred to nitrocellulose and immunoblotted with anti-ISG15 or anti-phosphoSer473**-**AKT. The expression of AKT was evaluated using a specific antibody. Actin was measured as protein loading control. Protein standards are indicated. **F–G** Quantification of phagocytic cell, expressed as relative amount of the total number of macrophages, and the viral titer was determined in the absence (**F**) or presence of 100 nM wortmannin (**G**). Means and standard deviations of a typical example from three independent experiments are presented. P values from a two-tailed t test assuming non-equal variance were determined. In all the cases, P<0.05.

As ISG15 plays a critical role in latex beads and virus entry in macrophages, we wanted to analyze if ISG15 also participates in the phagocytosis of infected cells by macrophages. A function of ISG15 in this process would also at least partially explain the high susceptibility of ISG15−/− mice to viral infections. To assess this possibility, ISG15−/− MEFS were infected with VACV-YFP at 1 PFU/cell for 8 hours, and subsequently added to macrophages cultures at a ratio of one MEF to four macrophages. The mixed culture was incubated for 2,5 hours at 37°C and the number of macrophages containing fluorescent signal was determined. When VACV-YFP-infected MEFs were mixed with ISG15+/+ peritoneal macrophages, efficient phagocytosis was observed (Fig. 9A). In contrast, a massive decrease in phagocytosis of infected cells was found when infected MEFs were mixed with ISG15−/− macrophages (Fig. 9A). Quantification of these results revealed that the phagocytic capacity of the ISG15+/+ macrophages was about 20 times higher than that observed in ISG15**−/−** (Fig. 9F). More than 40% of the ISG15+/+ macrophages incorporated virus-infected cells, whereas only about 2% of the ISG15−/− macrophages phagocyted VACV infected cells. These results show that in macrophages ISG15 is necessary to phagocytize VACV-infected cells. To study whether the phagocytosis is or not exclusively due to the macrophages in these co-cultures experiments, we decided monitored the phagocytosis capacity in MEFs using GFP-latex. A clear absence of latex bead was observed inside the cells indicating that MEFs do not exhibit phagocytic ability (Figure S4). However, although the MEFs were not able to phagocyte latex beads the infection with the VACV-YFP showed that virus entry occurred normally in these cells (Figure S4).

Phagocytosis of infected cells could contribute to the virus clearance and, if during this process the macrophage gets infected, it could commit suicide, blocking further possible propagation of the virus infection. Thus, we decided to examine whether virus growth in MEFs is affected in the presence of macrophages, as a simulation of macrophage mediated viral clearance. For this purpose, ISG15−/− MEFs were infected with VACV and added to a macrophage culture after 8 hpi. The co-culture was further maintained and the virus titer in the culture medium was determined at 24 and 48 h after infection. The virus titer in the infected cells of the co-cultures that contains ISG15 +/+ macrophages gradually decreased, while that in the control culture with DMEM and in the co-culture with ISG15−/− macrophages clearly increased the time (Fig. 9B). Furthermore, in the co-culture experiments with ISG15+/+ macrophages, the reduction in viral titer was accompanied with a reduction in VACV viral protein expression (Fig. 9C), an increase in ISG15 levels (conjugated and non-conjugated) (Fig. 9D) and a clear increase in the AKT phosphorylation level (Fig. 9-E). These results clearly indicate that the production of VACV into the cells is inhibited in the presence of high levels of ISG15 in macrophages and strongly suggest that this was due to phagocytosis of infected cells. However, the possibility remained that soluble factors, secreted from macrophages and could cause a decrease in viral replication.

To investigate the influence of cell-to-cell contact in these observations and to rule out the contribution of soluble antiviral molecules secreted by the macrophages in the above described results, we performed similar co-culture assays in which MEF and macrophages were physically separated in special plates but shared a common culture medium. When macrophages and infected cells were cultured in plates with two isolated compartments, no antiviral effect was observed in any case (Figure S5). These results indicate that direct contact between virus-infected cells and macrophages is required for the clearance of viral infection. This observation implies that cytokines or other soluble mediators, like secreted free ISG15 or IFNs, were not directly responsible for the ISG15 mediated antiviral effect of macrophages. Finally, to confirm that the ISG15-mediated phagocytic capacity regulates the viral clearance, we studied the effect of wortmannin treatment in virus clearance. ISG15−/− MEFS were infected with VACV-YFP at 1 PFU/cell for 8 hours, and added to the macrophages cultures, which were previously treated or not with wortmannin for 1 hour. The mixed culture was incubated for 2,5 hours at 37°C and the number of macrophages containing fluorescent signal was determined. When the infected MEFs were mixed with ISG15+/+ peritoneal macrophages, efficient phagocytosis was observed. As expected, intake of infected cells was completely abrogated when AKT phosphorylation was blocked by wortmannin treatment in the peritoneal macrophages, resembling the phenotype observed in ISG15−/− macrophages (Fig. 9F–G, upper panels). Moreover, and further confirming the role of AKT phosphorylation in the phagocytosis mediated viral clearance, the reduction of virus production was completely inhibited upon wortmannin treatment (Fig. 9F–G, lower panels), resembling the observation in co-culture experiments with ISG15−/− macrophages. These results indicate that phagocytosis of virus-infected cells and the subsequent virus clearance is strongly dependent on AKT phosphorylation. In summary, these studies established a novel mechanism by which ISG15 controls viral-induced macrophage phagocytosis and its antiviral activities through AKT dependent pathway in response to VACV infection.

## Discussion

The host innate immune response, including the production of type-I IFN, represents the primary line of defense against viral pathogens. Of the hundreds of IFN-stimulated genes (ISGs) discovered to date, *ISG15* was one of the firstly identified and shown to encode an ubiquitin-like protein modifier [34]. ISG15 knock-out mice are more susceptible to infection by several viruses, pointing out the relevance of this molecule in the antiviral response in animal models [35]. However, the underlying causes of the enhanced susceptibility of ISG15**−/−** mice and the cell types involved are only weakly defined. In order to clarify these questions, we evaluated the role of ISG15 in VACV replication in different cells types. Both MEFs and dendritic cells (not shown) VACV-infected were unaffected by the lack of ISG15 in the course of infection. In contrast, ISG15−/− peritoneal macrophages showed a clear resistance to VACV-induced cell death in comparison with wild type control macrophages where VACV replication is abortive and infectious progeny is not released [29]. Whereas WT macrophages became apoptotic and die after infection, in ISG15−/− macrophages, in which VACV infection is also abortive, no apoptosis was observed, as evidenced by the absence of PARP cleavage and CPE (Fig. 2). Apoptosis after infection with many types of viruses is generally considered as a self-defense mechanism [36], as loss of host cell activity should impair virus propagation. For instance, apoptotic cells are engulfed and digested in lysosomes of phagocytes [36]. Moreover, a higher increase in the expression of ISG15 and the protein ISGylation levels were observed in ISG15+/+ infected macrophages when compared to infected MEFs, indicating that the factor involved in the different phenotype among VACV infection in these cells could be modified by ISG15.

In addition to the differences in the VACV-induced apoptosis between ISG15+/+ or ISG15−/− macrophages, we observed clear differences in the phagocytosis ability of the macrophages. Apoptosis and phagocytosis are two interconnected pathways, which are very important for the efficacy of the innate immune response against pathogen infection [36]. Phagocytic elimination of invading microbes represents an important innate immune mechanism, as it contributes to the clearance of the virus. Moreover, if during this process the macrophage gets infected, it will commit suicide by apoptosis, blocking further possible propagation of the viral infection. In contrast, viruses appear to resist this host action by inhibiting apoptosis using anti-apoptotic proteins encoded by viral genes [37]. Moreover, the phagocytosis of apoptotic bodies seems to be an important mechanism to cross-prime DCs, as extracellular antigens gain access to MHC I molecules for priming of cytolytic T-cells [38]. Some microbes including viruses are phagocytized not directly but indirectly as microbe-infected cells [33]. Phagocytosis of immune complexes, opsonized virus, and infected host cells represents another important connection between the adaptive and innate immune systems, with potential roles both in priming of the adaptive immune response and in clearance of virus. Phagocytosis not only may rapidly remove virus or virally infected cells from the circulation but it could also affect immune complex-induced inflammation, which is implicated in driving disease progression. Importantly, there is evidence that disease susceptibility and severity in numerous autoimmune diseases and infectious diseases are regulated by phagocytosis [39]. While ISG15−/− mice were not more susceptible to VACV wild type virus infection, this might be explained by the capacity for VACV to inhibit IFN signaling and therefore ISG15 induction during infection in *vivo*. In fact, a VACV with enhanced IFN induction due to the deletion of *E3L* displayed higher pathogenicity in ISG15−/− mice [10]. It is attractive to speculate that the increased susceptibility of ISG15−/− mice to several virus infections, including herpesvirus and influenza virus, is mediated at least in part by a defect in macrophage phagocytosis. Further experimentation is required to demonstrate that this is the case.

As we mentioned before, our results reflect a diminution in the apoptosis and in the phagocytosis of ISG15−/− macrophages. Interestingly, type I IFN increases the phagocytic ability of macrophages in an ISG15 dependent manner, strongly suggesting that the ability of IFN to activate phagocytosis by macrophages is mediated by upregulation of ISG15.

The phosphatidylinositol 3-kinase (PI3K) pathway is an important signaling pathway that modulates diverse cellular activities, including cell survival, growth, proliferation, metabolism, migration, and apoptosis [40]. A great number of viruses utilize the PI3K-AKT cell signaling pathway to promote various steps in their replication cycle, such as the regulation of gene expression and the genome replication. Some bacteria and a few non-enveloped viruses also utilize this pathway to trigger their invasion and phagocytosis into cells [41]–[43]. Recently, it has been published that VACV induces AKT phosphorylation to allow viral entry in an integrin β1-dependent manner, suggesting that integrin β1-mediates PI3K/AKT activation induced by VACV [44]. Interestingly, when we examined the phagocytic capacity of ISG15−/− macrophages, the deficiency in the uptake of latex beads, VACV and VACV-infected MEFs was accompanied by a decrease in the phosphorylation levels of AKT. Moreover, the inhibition of the PI3K/AKT pathway in macrophages leaded to a reduction of phagocytosis and virus clearing capacity. These results further confirm that the initial transient activation of AKT is required for macrophages antiviral activities.

Currently, there is increasing evidence of the involvement of AKT pathway in the biological effects of IFNs [45]. In this sense, our data indicate a clear connection between this pathway and ISG15 in the control of phagocytosis and antiviral defense. In particular, we concluded that the regulation of the macrophage antiviral response by ISG15 is dependent on AKT phosphorylation. Consequently, our results suggest that the interconnection between IFN response and the AKT pathway could be at the base of the increased susceptibility to viral infection in the absence of ISG15. Optimal phagocytosis by macrophages is likely necessary to exert the antiviral action and to allow the clearance of the virus to take place. These mechanisms could be crucial for the ISG15 antiviral activity in animal models. Our results clearly indicate that a physical contact between the macrophages and the infected cells is required for antiviral effects, suggesting that intracellular ISG15 regulate this process. However, ISG15 is also secreted by neutrophils, monocytes and lymphocytes, and this released ISG15 controls T and natural killer (NK) lymphocytes, principal inductors of interferon (IFN)-γ [28]. In addition, the lack of secreted ISG15 is associated with severe mycobacterial disease in both mice and humans [28]. Taking into account that the intracellular survival of *Mycobacterium tuberculosis* depend on its ability to arrest phagolysosome biogenesis blocking the efficient antigen processing and presentation in macrophages [46], it is likely that this novel mechanism described here might also play a role in the increased susceptibility of ISG15 deficient humans to bacterial infections, although further investigations will be needed to evaluate this possibility.

In summary, we have identified a key pathway that links the macrophage phagocytosis capacity with a post-phagocytic signaling event. This, in turn, could be required for the macrophage-mediated antiviral activity essential for the virus clearance and the immune response following infection. These novel findings further underline the importance of ISG15 in the control of innate immunity. Further investigations on how these different processes are regulated by ISG15 and how these events affect downstream immune functions *in vivo* will be required to understand the role of ISG15 in the generation of tissue protective responses during infection with different pathogens.

## Materials and Methods

### Cells, viruses, and infections

ISG15−/− MEFs and their wild type counterpart were generated by Osiak et al. [13] and cultured in DMEM with 10% fetal calf serum (FCS). VACV wild-type Western Reserve strain (WR) was grown on monkey BSC-40 cells (African green monkey kidney cells, ATCC number CRL-2761), purified by sucrose gradient banding and titrated in BSC-40 cells as described [47]. VACV-YFP , a generous gift of Michael Way, was grown as described [48]. Influenza virus A/Wilson Smith N*(WSN)*/1933strain was grown on MDCK cells (ATCC number CCL-34) and titrated in MDCK cells as described [49].

### ISG15+/+ or ISG15−/− peritoneal macrophages isolation

Resident peritoneal macrophages were isolated from mice by peritoneal lavage using 10 ml DMEM. Lavage fluid was centrifuged (500× g, 5 min) and cells were cultured in Petri dishes in DMEM containing 10% fetal bovine serum, 100 U/ml penicillin and 100 µg/ml streptomycin (3 h, 37°C, 5% CO2). Non-adherent cells were removed by extensive washing with DMEM as described [50], this protocol showed that the purity of peritoneal macrophage was ≥95% and their immunophenotype profile was typical of F4/80 positive (see Figure S1). All animals were handled in strict accordance with good animal practice as defined by the relevant national, international, and/or local animal welfare bodies, and with the Spanish Royal Decree (RD 1201/2005). All animal work was approved by the Ethical Committee of Animal Experimentation (CEEA-CNB) of the Centro Nacional de Biotecnología (CNB-CSIC).

### Cellular viability assay

Cells were grown to confluence in 96-well plates and infected with VACV virus at the indicated multiplicity of infection (MOI) from 0.01 to 10 PFU/cell. 24 hours post-infection (hpi), the medium was removed and cytolysis was determined by crystal violet staining as described previously [51]. The percentage of viable cells after infection was calculated assuming the survival rate of uninfected cells to be 100%.

### Western blot analysis

ISG15+/+ and ISG15**−/−** fibroblast or peritoneal macrophages were infected (10^6^ cells/time post-infection; 10 PFU/cell) with VACV and collected at the indicated times post-infection (0, 2, 6, and 16 hpi). Cell extracts were obtained using lysis buffer (50 mM Tris**-**HCl, 0.5 M NaCl, 10% NP**-**40, 1% SDS) and protein extraction was performed by 5 min incubation on ice. Protein lysates (100 µg) were fractionated by 14% or 8% SDS-PAGE, transferred to nitrocellulose membranes, and incubated with anti**-**ISG15 [12], anti**-**tubuline (Sigma), anti**-**AKT (Cell signaling), anti-phosphoS473-AKT (Cell signaling), anti-caspase 3 (Oncogene), anti-caspase 9 (Oncogene), anti-bcl2 (Santa Cruz), anti-eIF-2α (Santa Cruz), anti-phospho-T202/Y204- Erk1/2 (Santacruz, kindly provided by S. Alemany) anti-anti Erk1/2 (Santa Cruz), anti-anti-S35-eIF2α (Invitrogen) and anti-PARP (Cell Signaling) antibodies, followed by secondary antibodies (mouse and rabbit peroxidase conjugates from Sigma). Protein expression was detected using ECL reagents (Amersham).

### Analysis of ^35^S-methionine labeled proteins

ISG15+/+ and ISG15−/− fibroblast or peritoneal macrophages were infected (10^6^ cells/time postinfection; 3 PFU/cell) with VACV (Western Reserve strain) or FluV (WSN, *A/WSN/1933*). At indicated hpi, cells were washed with methionine-free medium and incubated with methionine-free medium containing 50 µCi/ml of ^35^S-methionine (30 min, 37°C). Cells extracts were prepared as described in lysis buffer (50 mMTris-HCl, 0.5 M NaCl, 10% NP-40, 1% sodium dodecyl sulfate [SDS]), fractionated by 12% SDS- polyacrylamide gel electrophoresis (PAGE) and developed by autoradiography.

### Determination of caspase 3/7 activation

Apoptosis quantification was carried out using the Caspase-Glo 3/7 assay kit (Promega), following the protocol recommended by the supplier. Briefly, ISG15+/+ and ISG15−/− fibroblast or peritoneal macrophages monolayers grown in 96 well plates were infected at the indicated MOI. At the specified times post-infection, 100 µl of Caspase-Glo 3/7 reagent was added to the wells under study. Plates were gently shaken and then incubated in the dark at 20°C for 60 min before recording the luciferase activity using an Orion microplate luminometer (Berthold technologies).

### Latex bead phagocytosis

ISG15+/+ and ISG15**−/−** peritoneal macrophages or MEFS were seeded in 8**-**well μ-Slide microscopy chambers (Ibidi, Germany) and filmed after the incubation with 1**-**µm-diameter latex beads conjugated to green fluorescent protein (GFP) (Sigma), in a ratio of 10 beads per cell. Alternatively cells were cultured on coverslips and incubated for 1 h with the beads (20 or 10 beads per cell, depending of the experiment), washed three times with PBS and incubated with DMEM medium for an additional hour as described [31]. Cells were fixed with 4% PFA and processed for immunofluorescence analysis. Briefly, cells were washed with phosphate**-**buffered saline (PBS), fixed with 4% paraformaldehyde, and permeabilized with 0.1% Triton X**-**100 in PBS (room temperature, 10 min), DNA was stained with ToPro 3 (Life Technologies). Images were obtained using a Bio-Rad Radiance 2100 confocal laser microscope.

### Phagocytosis of VACV-infected MEFs by macrophages

The phagocytosis assay with mouse peritoneal macrophages was conducted as previously described [52]. ISG15+/+ and ISG15**−/−** peritoneal macrophages were seeded in 8**-**well μ-Slide microscopy chambers (Ibidi, Germany) during 48 hours and washed to remove the non-adherent cells. ISG15−/− MEFS were infected with VACV-YFP at 1 PFU/cell for 14 hours, and added to the macrophages culture at ratio of one MEF cells to four macrophages. The mixed culture was incubated for two hours at 37°C and the number of macrophages contained fluorescent signal was determined and expressed relative to the total number of macrophages as the phagocytic index.

### Live cell imaging

Images from live ISG15+/+ or ISG15**−/−** macrophages or MEFS infected with VACV-YFP or treated with latex beads were collected every 10 minutes for 2 hours using a Leica Plan APO 20×/0.70 objective mounted on a Leica TCS SP5 confocal system as described previously [53].

## Supporting Information

Figure S1 **Presence of macrophages in murine peritoneal lavages.** ISG15+/+ or ISG15−/− peritoneal macrophages were cultured on coverslips and incubated for 72 h, non-adherent cells were removed by extensive washing with DMEM. Cells were fixed with 4% PFA and processed for immunofluorescence analysis using an anti-F4/80 specific AB. Representative fields are shown at a magnification of 40×.
(TIF)

Figure S2 **Restrictive infection of peritoneal macrophages ISG15+/+ or ISG15−/− to influenza virus.** ISG15+/+ or ISG15**−/−** macrophages were infected (10^6^ cells/time post-infection; 3 PFU/cell) with FluV (A/*WSN*/1933 strain) and at the different times indicated, cells were harvested and virus yields were determined by plaque assay. Results represent the mean ± the standard deviation of three independent experiments. P values from a two-tailed t test assuming non-equal variance were determined. In all the cases, P<0.01.
(TIF)

Figure S3 **IFN treatment enhances the VACV entry in macrophages.** ISG15+/+ and ISG15**−/−** peritoneal macrophages were treated with type I IFN alpha (100 units/ml for 16 hours) or left untreated and infected with VACV (60 PFU/cell) and at 2 hours postinfection cells were fixed with PFA 4%, washed one in PBS, permeabilized and labeled with anti-WR antibodies, followed by the appropriate fluorescent secondary AB and ToPro reagent. The cells were analyzed by confocal immunofluorescence microscopy. Representative fields are shown at a magnification of 100×.
(TIF)

Figure S4 ***In vivo*** **imaging of latex beads phagocytosis and VACV-YFP infection in MEFs.** **A.** Phagocytosis of GFP-latex beads by ISG15 +/+ MEFs. Cells were seeded in 8-well tissue culture plates and treated with type I IFN (100units/ml) for 16 hours. After that, the cells were incubated with 1**-**µm-diameter latex beads conjugated to GFP in a ratio of 10 latex beads per cell. Phagocytized beads and cells were visualized with the time by fluorescent and phase contrast microscopy. Representative fields are shown at a magnification of 40× (left panels). **B.** MEFs isolated from ISG15+/+ mice were seeded in 8-well tissue culture plates and treated with type I IFN (100units/ml) for 16 hours. After that, the cells infected with VACV- YFP (60 PFU/cell). Infected-cells were visualized with the time by fluorescent and phase contrast microscopy. Representative fields are shown at a magnification of 40× (left panels).
(TIF)

Figure S5 **ISG15 requires direct contact between virus-infected cells and macrophages for the regulation of the phagocytosis and the clearance of VACV-infected MEFS.** ISG15−/− MEFS were infected with VACV-YFP at 1 PFU/cell for 8 hours, and were co-cultivated in a 2 chambers plate with DMEM (as negative control) or macrophages (ISG15+/+ or ISG15**−/−**). Infected cells were harvested at different times postinfection and virus yields were determined by plaque assay. Results represent the mean ± the standard deviation of three independent experiments. P values from a two-tailed t test assuming non-equal variance were determined. In all the cases, P<0.05.
(TIF)

Movie S1 **The movie shows the CPE produced after VACV infection in ISG15+/+ macrophages.** The film started at 8 hpi and finished at 9 hpi (time stamp indicates hours and minutes).
(AVI)

Movie S2 **The movie shows the CPE produced after VACV infection in ISG15−/− macrophages. **The film started at 8 hpi and finished at 9 hpi (time stamp indicates hours and minutes).
(AVI)

Movie S3 **The movie shows the phagocytosis produced after the incubation of ISG15+/+ macrophages with GFP-labeled latex beads. **The film started at t = 0 and finishes 2 hours later.
(AVI)

Movie S4 **The movie shows the phagocytosis produced after the incubation of ISG15−/− macrophages with GFP-labeled latex beads. **The film started at t = 0 and finishes 2 hours later.
(AVI)

Movie S5 **The movie shows the phagocytosis produced after the incubation of IFN treated ISG15+/+ macrophages with GFP-labeled latex beads. **The film started at t = 0 and finishes 2 hours later.
(AVI)

Movie S6 **The movie shows the phagocytosis produced after the incubation of IFN treated ISG15−/− macrophages with GFP-labeled latex beads. **The film started at t = 0 and finishes 2 hours later.
(AVI)

Movie S7 **The movie shows the phagocytosis produced after the incubation of ISG15+/+ macrophages IFN treated with GFP-labeled latex beads. **The film started at t = 0 and finishes 2 hours later.
(AVI)

Movie S8 **The movie shows the phagocytosis produced after the incubation of wortmanin treated (100 nM) ISG15+/+ IFN exposed macrophages with GFP-labeled latex beads.** The film started at t = 0 and finishes 2 hours later.
(AVI)

Movie S9 **The movie shows the phagocytosis produced after the incubation of rapamycin treated (100 nM) ISG15+/+ IFN exposed macrophages with GFP-labeled latex beads. **The film started at t = 0 and finishes 2 hours later.
(AVI)

Movie S10 **The movie shows the phagocytosis produced after the incubation of LPS treated (100 nM) ISG15+/+ macrophages with GFP-labeled latex beads. **The film started at t = 0 and finishes 2 hours later.
(AVI)
