# Correction: ISG15 Regulates Peritoneal Macrophages Functionality against Viral Infection

**DOI:** 10.1371/annotation/64a20cef-bf55-4d84-983e-b914eb09b1ee

**Published:** 2013-10-30

**Authors:** Emilio Yángüez, Alicia García-Culebras, Aldo Frau, Catalina Llompart, Klaus-Peter Knobeloch, Sylvia Gutierrez-Erlandsson, Adolfo García-Sastre, Mariano Esteban, Amelia Nieto, Susana Guerra

There is an error in the affiliation for author Emilio Yángüez. The affiliation where the work was completed is the following:

Department of Molecular and Cellular Biology, Centro Nacional de Biotecnología CSIC, Madrid, Spain

The current affiliation for Emilio Yángüez is: Institute of Medical Virology, University of Zurich, Zurich, Switzerland 

