# Correction: ISG15 Regulates Peritoneal Macrophages Functionality against Viral Infection

**DOI:** 10.1371/journal.ppat.1005969

**Published:** 2016-10-13

**Authors:** Emilio Yángüez, Alicia García-Culebras, Aldo Frau, Catalina Llompart, Klaus-Peter Knobeloch, Sylvia Gutierrez-Erlandsson, Adolfo García-Sastre, Mariano Esteban, Amelia Nieto, Susana Guerra

There are errors in the Funding section. The correct funding information is as follow: This work was supported by grants from a FIS grant PI11/00127 (Fondo de Investigación Sanitaria del Instituto de Salud Carlos III and Ministry of Health of Spain, State secretary of R+D and FEDER/FSE), Comunidad de Madrid UAM-CM-CCG10-4911 and UAM-Banco de Santander to SG. This work was also partly supported by NIAID grant U19AI083025 to AG-S. The funders had no role in study design, data collection and analysis, decision to publish, or preparation of the manuscript.
