# Supplementary figures and images for: ISG15 Regulates Peritoneal Macrophages Functionality against Viral Infection
Source: PLoS Pathog. 2013 Oct 10;9(10):e1003632. doi: 10.1371/journal.ppat.1003632 (PMC3796851; doi:10.1371/journal.ppat.1003632)

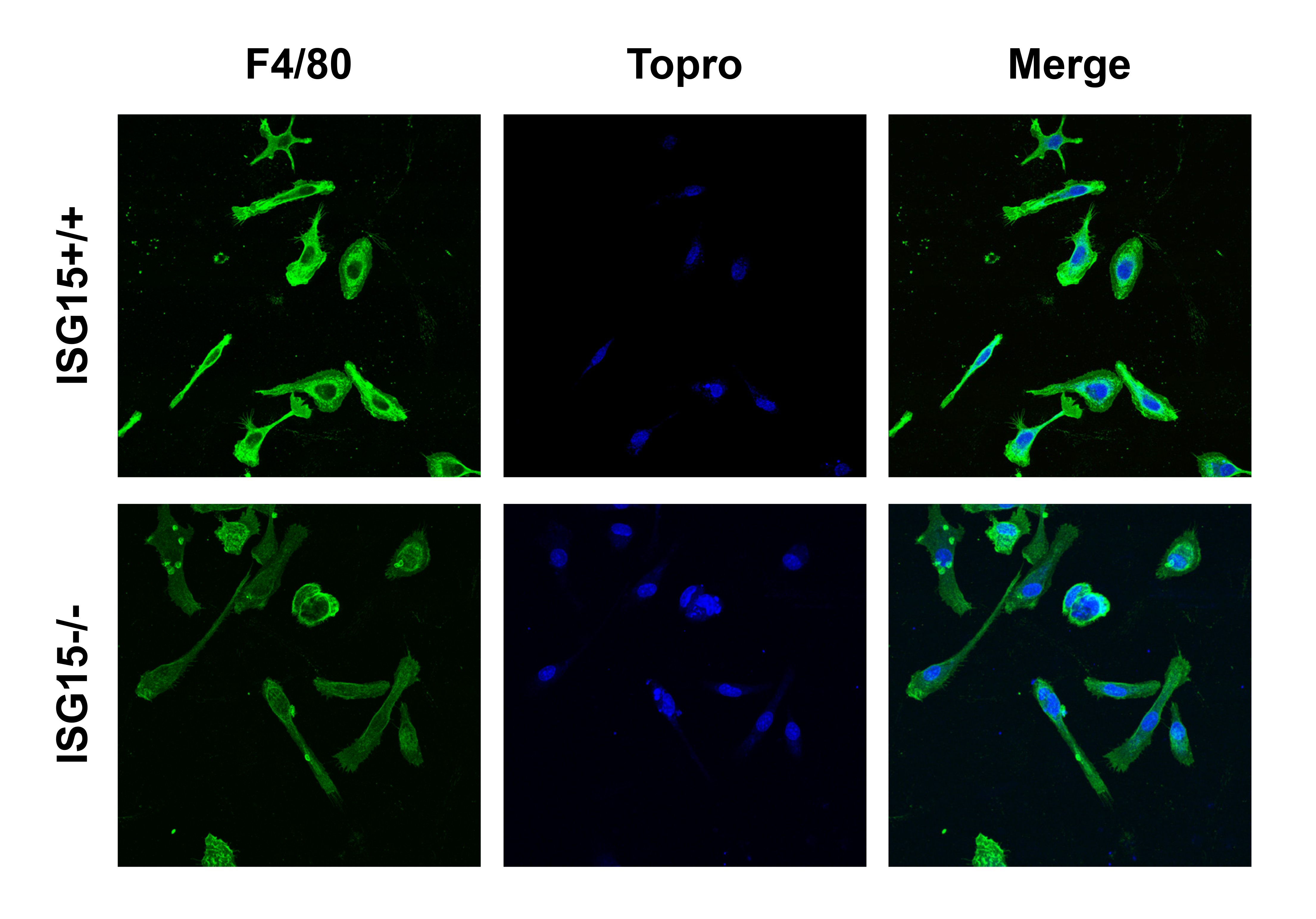

Supplement: Figure S1 — Presence of macrophages in murine peritoneal lavages. ISG15+/+ or ISG15−/− peritoneal macrophages were cultured on coverslips and incubated for 72 h, non-adherent cells were removed by extensive washing with DMEM. Cells were fixed with 4% PFA and processed for immunofluorescence analysis using an anti-F4/80 specific AB. Representative fields are shown at a magnification of 40×. (TIF) [file ppat.1003632.s001.tif]

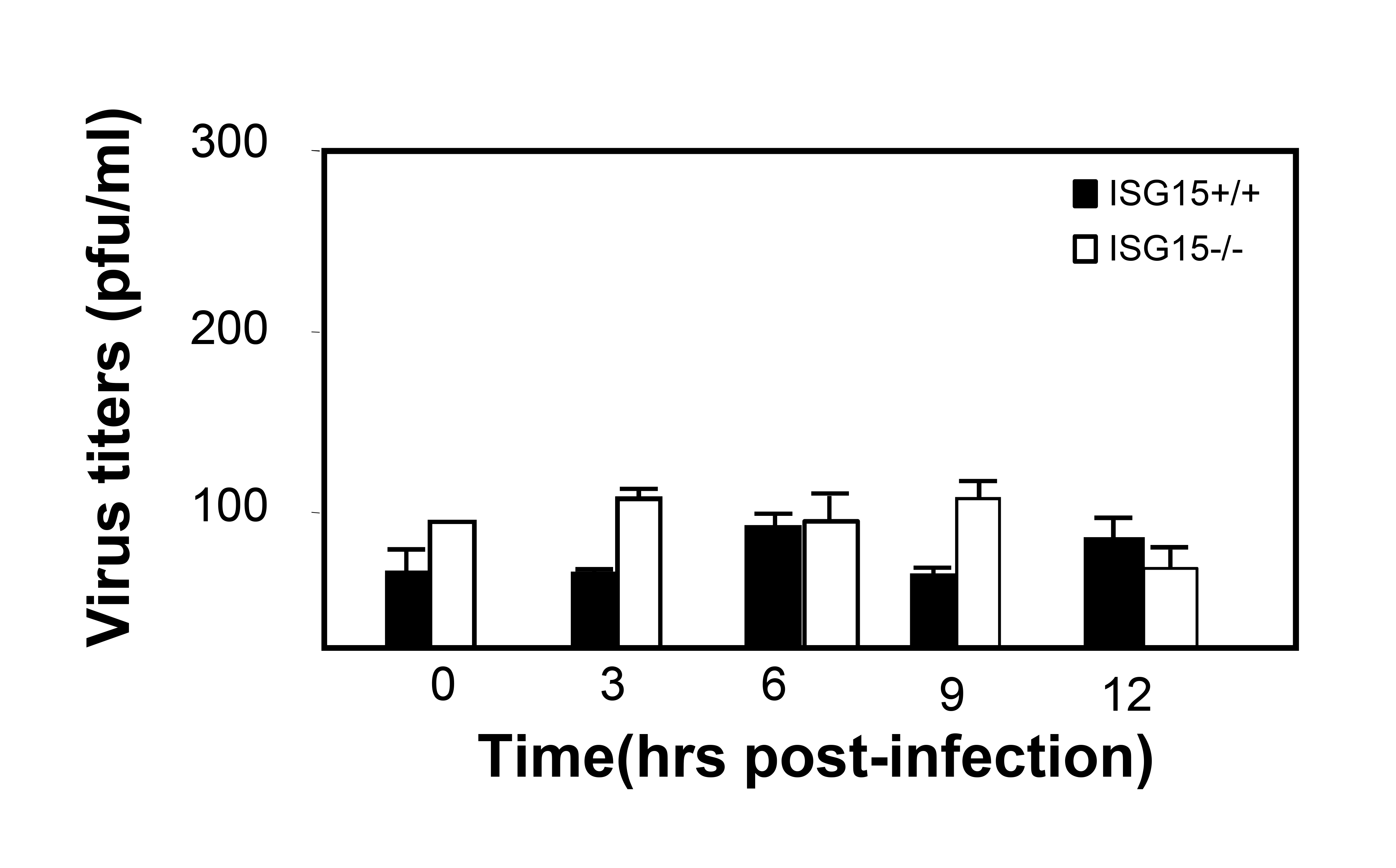

Supplement: Figure S2 — Restrictive infection of peritoneal macrophages ISG15+/+ or ISG15−/− to influenza virus. ISG15+/+ or ISG15−/− macrophages were infected (106 cells/time post-infection; 3 PFU/cell) with FluV (A/WSN/1933 strain) and at the different times indicated, cells were harvested and virus yields were determined by plaque assay. Results represent the mean ± the standard deviation of three independent experiments. P values from a two-tailed t test assuming non-equal variance were determined. In all the cases, P<0.01. (TIF) [file ppat.1003632.s002.tif]

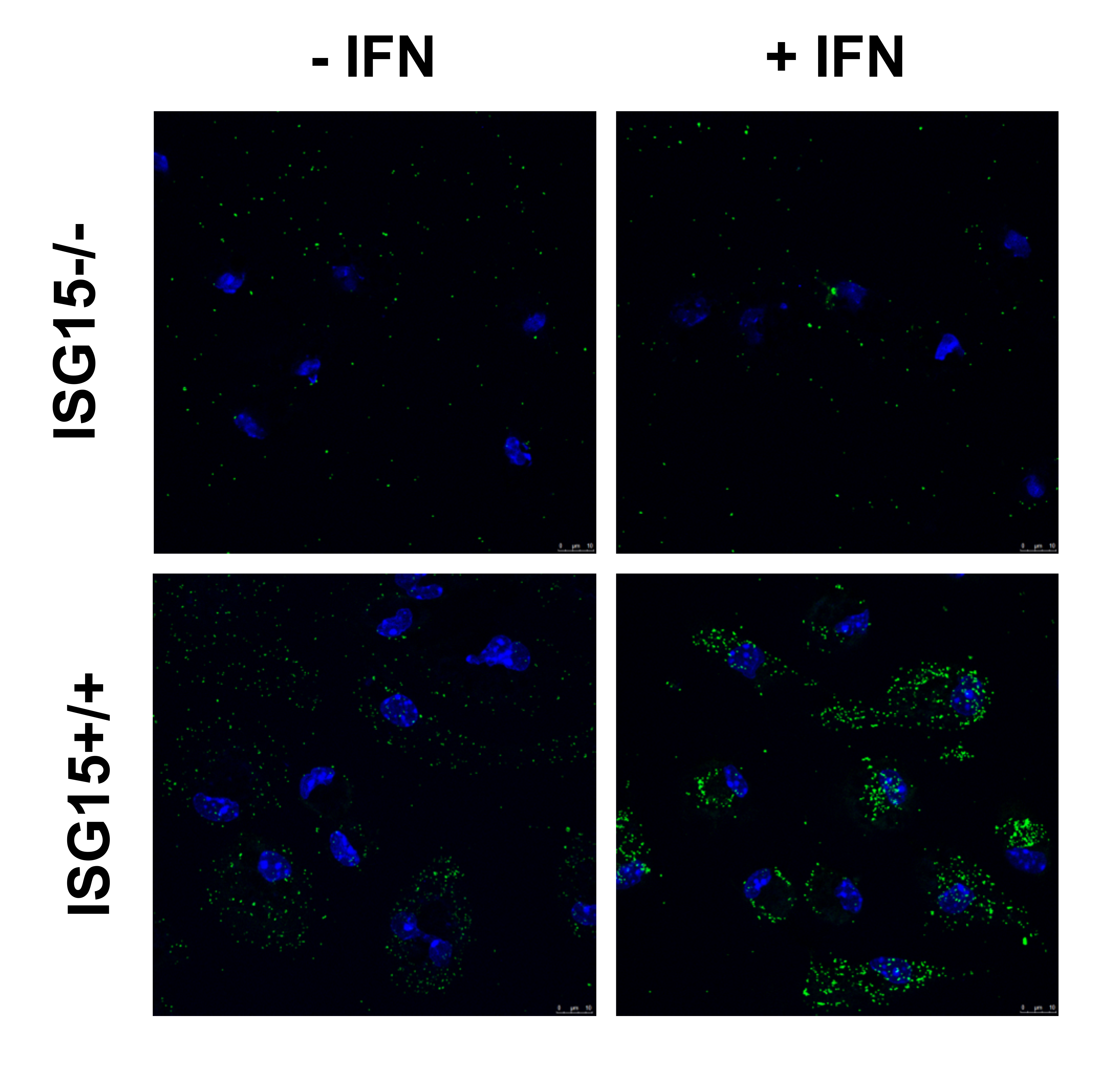

Supplement: Figure S3 — IFN treatment enhances the VACV entry in macrophages. ISG15+/+ and ISG15−/− peritoneal macrophages were treated with type I IFN alpha (100 units/ml for 16 hours) or left untreated and infected with VACV (60 PFU/cell) and at 2 hours postinfection cells were fixed with PFA 4%, washed one in PBS, permeabilized and labeled with anti-WR antibodies, followed by the appropriate fluorescent secondary AB and ToPro reagent. The cells were analyzed by confocal immunofluorescence microscopy. Representative fields are shown at a magnification of 100×. (TIF) [file ppat.1003632.s003.tif]

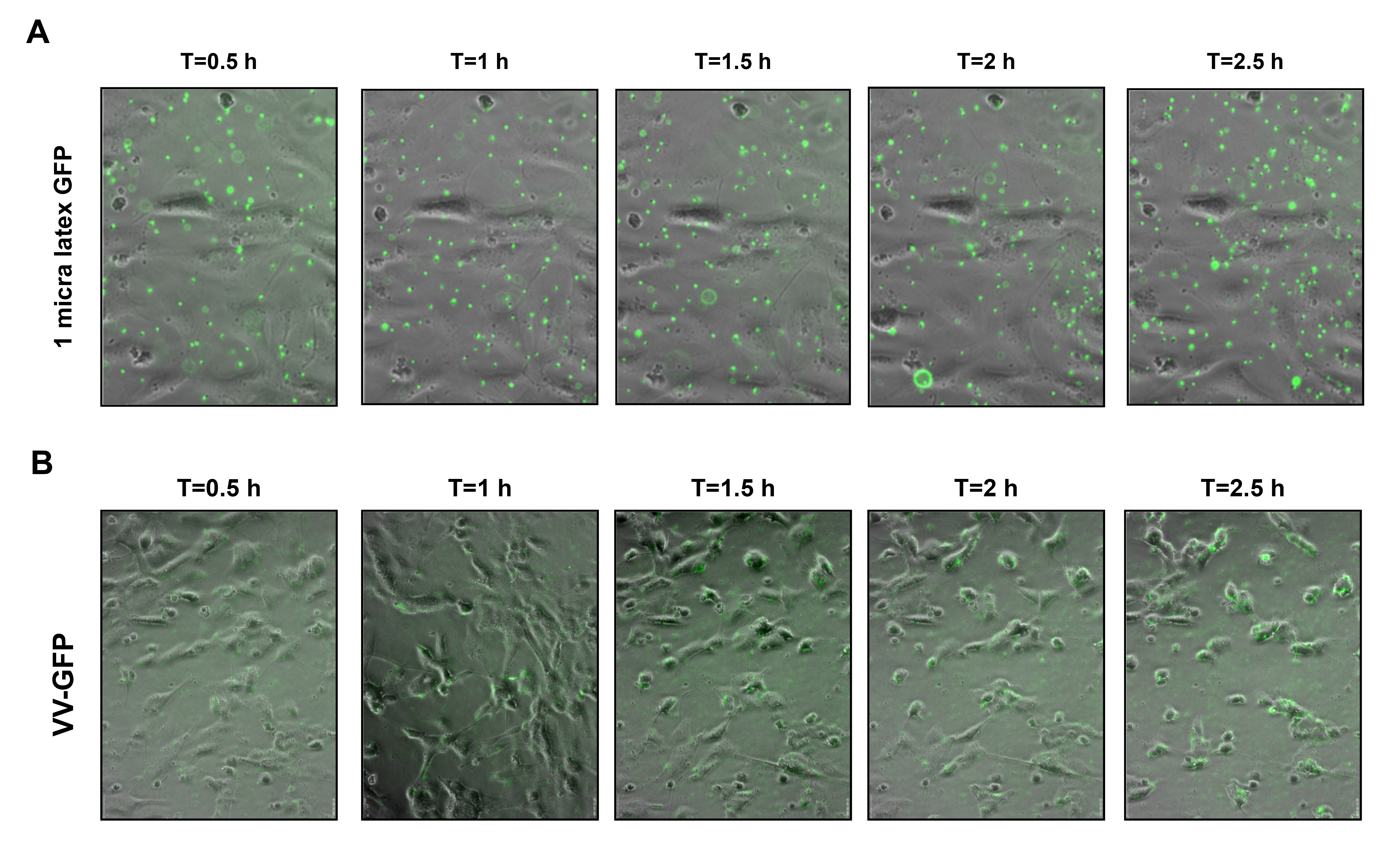

Supplement: Figure S4 — In vivo imaging of latex beads phagocytosis and VACV-YFP infection in MEFs. A. Phagocytosis of GFP-latex beads by ISG15 +/+ MEFs. Cells were seeded in 8-well tissue culture plates and treated with type I IFN (100units/ml) for 16 hours. After that, the cells were incubated with 1-µm-diameter latex beads conjugated to GFP in a ratio of 10 latex beads per cell. Phagocytized beads and cells were visualized with the time by fluorescent and phase contrast microscopy. Representative fields are shown at a magnification of 40× (left panels). B. MEFs isolated from ISG15+/+ mice were seeded in 8-well tissue culture plates and treated with type I IFN (100units/ml) for 16 hours. After that, the cells infected with VACV- YFP (60 PFU/cell). Infected-cells were visualized with the time by fluorescent and phase contrast microscopy. Representative fields are shown at a magnification of 40× (left panels). (TIF) [file ppat.1003632.s004.tif]

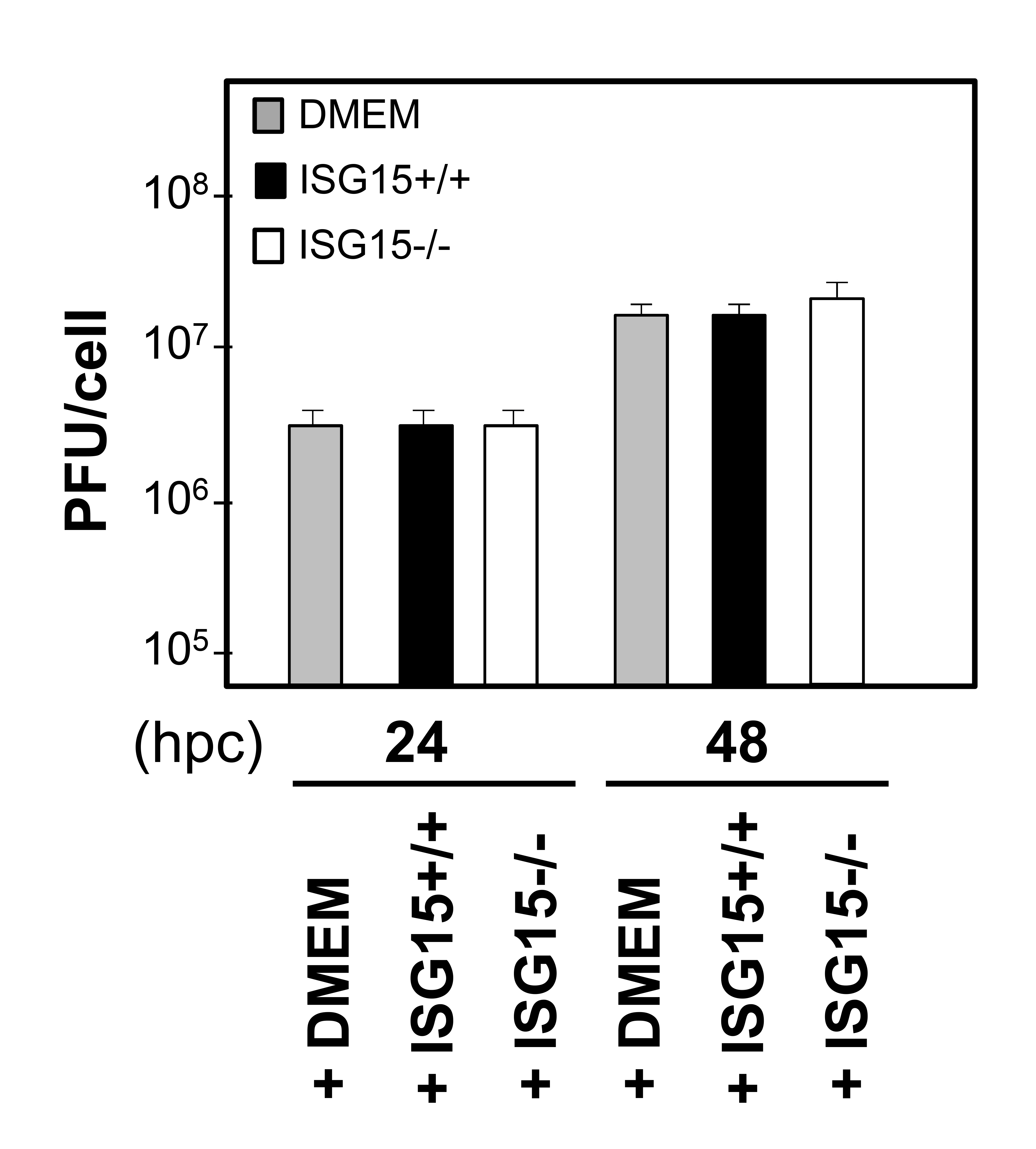

Supplement: Figure S5 — ISG15 requires direct contact between virus-infected cells and macrophages for the regulation of the phagocytosis and the clearance of VACV-infected MEFS. ISG15−/− MEFS were infected with VACV-YFP at 1 PFU/cell for 8 hours, and were co-cultivated in a 2 chambers plate with DMEM (as negative control) or macrophages (ISG15+/+ or ISG15−/−). Infected cells were harvested at different times postinfection and virus yields were determined by plaque assay. Results represent the mean ± the standard deviation of three independent experiments. P values from a two-tailed t test assuming non-equal variance were determined. In all the cases, P<0.05. (TIF) [file ppat.1003632.s005.tif]
